# The effectiveness and tolerability of trauma‐focused psychotherapies for psychotic symptoms: A systematic review of trauma‐focused psychotherapies

**DOI:** 10.1002/mpr.2005

**Published:** 2024-03-05

**Authors:** Jordan Reid, Charles Cole, Nabeela Malik, Vaughan Bell, Michael Bloomfield

**Affiliations:** ^1^ Translational Psychiatry Research Group Division of Psychiatry Research Department of Mental Health Neuroscience Institute of Mental Health University College London London UK; ^2^ Department of Clinical, Educational and Health Psychology University College London London UK; ^3^ University of Hertfordshire Hatfield UK

**Keywords:** psychosis, psychotherapy, PTSD, schizophrenia, trauma

## Abstract

**Introduction:**

Psychological trauma is an established risk factor for psychosis. Trauma‐focused psychotherapies (TFPT) have been suggested as a potential treatment for reducing psychotic symptoms in those who have experienced trauma. We therefore sought to investigate the effectiveness, tolerability, and acceptability of TFPT for psychotic symptoms.

**Methods:**

We conducted a systematic review of studies of any form of TFPT that measured psychotic symptoms across a broad range of diagnoses.

**Results:**

From 2584 papers initially identified, 17 studies (857 participants) met eligibility criteria. TFPT were found to be well tolerated, with very few adverse events. Acceptability was also high, with a mean dropout rate of 20%.

**Conclusions:**

Whilst the evidence of effectiveness for TFPT in reducing psychotic symptoms is weak, we found tentative evidence in favour of exposure‐based interventions. Methodologically rigorous trials investigating the efficacy of TFPT for the treatment of psychotic symptoms are needed to assess this promising intervention.

## 1 INTRODUCTION

### 1.1 Psychotic symptoms

Psychotic symptoms are characterised by delusions, hallucinations, and paranoia (positive symptoms) as well as difficulties with thinking, blunted emotions, and low motivation (negative symptoms) (Jablensky, 2010). Psychotic symptoms can occur in a range of disorders including schizophrenia spectrum disorders, bipolar disorder, major depressive disorder, dissociative disorders and borderline personality disorder and can therefore be considered a transdiagnostic phenomenon (Buckley et al., 2008). The life expectancy of people with psychotic symptoms is significantly reduced (Saha et al., 2007) due to suicide and greater health problems (Hannerz et al., 2001; Yuen et al., 2014).

### 1.2 Psychosis and trauma

Exposure to psychological trauma has consistently been associated with increased risk of psychotic experiences. The evidence fulfils the Bradford Hill criteria (Hill, 1965) supporting the hypothesis of a causative relationship between trauma and psychosis, including temporal relationships (Kelleher et al., 2013), dose‐response relationships (Croft et al., 2019) and plausible biological mechanisms (Howes & Murray, 2014).

Traumatic experiences can also give rise to post‐traumatic stress disorder (PTSD), and there is a growing body of evidence showing a relationship between PTSD and psychotic symptoms in people with psychosis who have experienced trauma (Bloomfield et al., 2021). PTSD is a risk factor for the development of psychotic symptoms (Okkels et al., 2016), and around 39% of people with psychosis experience concurrent PTSD (Mueser et al., 2010). Within trauma survivors, auditory hallucinations have been proposed to be a type of posttraumatic intrusion related to a traumatic memory (Peach et al., 2018; Steel, 2015). This is consistent with studies that have shown that hallucinatory content is often linked to experiences of trauma (Hardy et al., 2005; Onyeama et al., 2011; Peach et al., 2020). Within this framework, an intrusive trauma memory may not be experienced as a memory and is instead misattributed in a psychotic way (e.g. as a voice). Indeed, there are broad similarities between dominant cognitive theories of PTSD (Ehlers & Clark, 2000) and trauma‐induced psychotic symptoms (Morrison et al., 2003).

### 1.3 Trauma‐focused psychotherapies

Given the links between traumatic experiences, psychotic symptoms and PTSD, there is growing interest in trauma‐focused psychotherapies (TFPT) for psychotic symptoms. TFPT are a family of therapies developed to treat PTSD that explicitly focus on reprocessing memories of traumatic experiences (Schnurr, 2017). Some TFPT may utilise cognitive techniques only, some may use exposure, and some may use a combination of the two. Broadly, TFPT that utilise exposure are thought to work by promoting emotional habituation and reprocessing of traumatic memories via repeated exposure to the traumatic event and related cues. Cognitive therapies such as trauma‐focused CBT (TFCBT) additionally explicitly focus on restructuring peri‐traumatic (such as ‘I'm going to die’) and post‐traumatic (such as ‘I should have coped better’) appraisals, often utilising learnings gained from exposure. In the UK, National Institute for Clinical Excellence guidelines recommend the use of TFCBT and eye movement desensitisation and reprocessing (EMDR) in the treatment of PTSD (National Institute for Health and Clinical Excellence, 2018). Though the literature remains in its infancy, two recent reviews found that TFPT can safely reduce PTSD symptoms in those with psychosis (Sin & Spain, 2016; Swan et al., 2017).

### 1.4 The need for improved research and outcomes

There is little research into whether TFPT can reduce psychotic symptoms themselves. The only review to date (Brand et al., 2018) found small effects for TFPT on positive symptoms of psychosis. There remains therefore a pressing need for more research to improve treatments and outcomes for people with psychotic symptoms and trauma histories. We sought to address this by systematically reviewing the current evidence for the efficacy, tolerability, and acceptability of TFPT on psychotic symptoms. To differentiate this review from previously published work and to draw together disparate research, we have not limited the review to any specific type of TFPT. Additionally, as psychotic symptoms in the context of trauma are likely a transdiagnostic phenomenon (Buckley et al., 2008), we have not limited the review to any specific diagnosis.

## 2 MATERIALS & METHODS

### 2.1 Search strategy and selection criteria

We pre‐registered our review with PROSPERO (CRD42020202135). We followed the preferred reporting items for systematic reviews and meta‐analysis guidelines (PRISMA) (Moher et al., 2009). We included any study of the efficacy of a TFPT with a quantitative outcome measure for psychotic symptoms. We defined TFPTs as any psychological intervention from any modality of psychotherapy that had an explicit focus on past traumatic memories and/or was described as ‘trauma‐focused’. We distinguished between TFCBT that would be in‐line with the principles of Ehlers & Clark (2000) by facilitating memory reprocessing through exposure, and trauma‐informed CBT (TICBT) that did not include exposure. Interventions could be delivered in any setting in a group or individual format. We defined psychotic symptoms as hallucinations, delusions and/or paranoia.

We included studies on adults over the age of 18. Otherwise, we placed no limits on the population to be included. We included any study design that offered a TFPT, including case reports, case series and randomised controlled trials. We did not limit our criteria to any specific diagnoses. For example, dissociative identity disorder was included due to research highlighting elevated levels of psychotic symptoms (Foote & Park, 2008). We excluded studies that were not reported in English, were not published in peer‐reviewed journals, and studies of non‐clinical populations.

We used Ovid to systematically search medical and psychological databases (MEDLINE and PsycINFO) and ProQuest to search PTSDPubs from the earliest possible date to the date of the search. We searched a psychotic symptoms term (e.g. hallucinat* OR delus*) AND a trauma‐focused term (e.g. trauma* OR neglect*) AND a psychotherapy term (e.g. ‘Exposure therapy’ OR EMDR). See Supporting Information S1 for the full list of search terms by database and screening methodology.

Reviewer 1 (R1) imported articles generated from the search into a reference management software (EndNote 20) (The EndNote Team, 2013) and checked for duplicates which were removed from the review. R1 and reviewer 2 (R2) then screened for inclusion by title and abstract first, then by reading the full text, with any disagreements resolved by discussion, facilitated through the Rayyan platform (Ouzzani et al., 2016).

### 2.2 Data analysis

We defined the primary outcome a priori as quantitatively measured psychotic symptoms, including global measures of psychotic symptoms and measures of hallucinations, delusions, or paranoia. Our secondary outcomes were measures of other domains of psychopathology (depression, anxiety and PTSD) and social functioning. We assigned a level of evidence (OCEBM Levels of Evidence Working Group, 2011) with case reports assigned a level of evidence of 5. For randomised controlled trials (RCT), we assessed risk of bias using the Cochrane Risk of Bias 2 tool (Sterne et al., 2019). For case series, we used the Quality Appraisal Tool for Case Series Studies (Institute of Health Economics, 2014). For case reports, we used the Joanna Briggs Institute Checklist for Case Reports (Moola et al., 2017). R1 and reviewer 3 (R3) undertook a risk of bias and quality assessment. Any discrepancies between reviewers were resolved through discussion and by consensus. If agreement could not be reached, reviewer 5 (R5) was consulted to resolve this.

We extracted data manually from each study paper. R1 extracted the following data for each study: study design, *n*, participant characteristics and clinical presentation, location and setting of the treatment, the type of trauma participants had experienced, intervention type and dose, medications prescribed to participants, control or comparison, primary outcomes, secondary outcomes, adverse events (identified as adverse events reported by the authors and/or symptom exacerbation) and dropout rates. We grouped studies by therapeutic modality.

We considered findings statistically significant when the *p*‐value was below 0.05. Where possible, we calculated an effect size (Hedges' *g*; see Supporting Information S1 for formulae) for each study at each time point for each outcome of interest. We chose Hedges' *g* as it has superior properties to Cohen's *d* with small sample sizes (Cumming, 2012).

We undertook a narrative synthesis of quantitative outcomes following SWiM (synthesis without meta‐analysis) guidance (Campbell et al., 2020), grouping by modality of psychotherapy, including any relevant information about tolerability or acceptability of these interventions. We followed the GRADE guidance for clinical recommendations (Guyatt et al., 2008).

## 3 RESULTS

**FIGURE 1:**
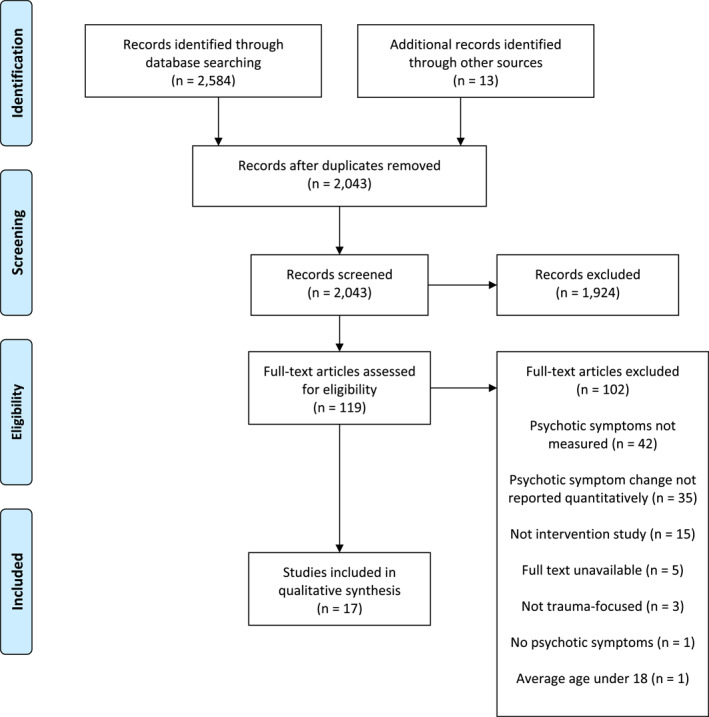
Preferred reporting items for systematic reviews and meta‐analyses flowchart.

We screened 2597 studies, identifying 17 studies (*n* = 857) that quantitatively measured psychotic symptoms in adults undergoing TFPT. Details of the selection process and exclusions at each stage are presented in the PRISMA flowchart (Figure 1; Moher et al., 2009). Table 1 provides a summary of the characteristics, primary outcomes, secondary outcomes, and adverse events of each study.

**TABLE 1:** Summary of the characteristics and outcomes of included studies.

Author	Design (level of evidence), *n*	Population, including diagnosis, location and setting	Treatment and dose	Primary outcomes	Secondary outcomes	Adverse effects	Dropouts
TICBT							
Mueser et al. (2015)	Multicentre RCT (1b) *n* = 201	Diagnoses of schizophrenia, schizoaffective disorder, major depression or bipolar disorder (DSM‐IV criteria); plus diagnosis of severe PTSD (based on CAPS). USA; 3 inpatient services and 2 outpatient services.	TICBT (breathing retraining, psychoeducation and cognitive restructuring) Minimum: 6 Mean: NR Maximum: 16 Length of time: NR	**Linear regression (across post‐treatment, 6 and 12 months):** **PANSS:** **Table:** Baseline mean Post‐treatment mean TICBT 65.75 62.25 Brief T 67.18 61.33 Non statistically significant decrease in TICBT group as compared to brief treatment across post‐treatment, 6 and 12‐month	**Linear regression (across post‐treatment, 6 and 12 months):** **CAPS:** Significant decrease in TICBT group as compared to brief treatment across post‐treatment, 6 and 12‐month: *F* = 6.51, (*p* = 0.01), Hedges' *g* = −0.29 Hedges' *g* of CBT versus brief treatment following treatment: −0.26 Hedges' *g* of TICBT versus brief treatment at 6‐month: −0.25 Hedges' *g* of CBT versus brief treatment at 12‐month: −0.19 **BDI‐II:** Non statistically significant increase in TICBT group as compared to brief treatment across post‐treatment, 6 and 12‐month **BAI:** Non statistically significant decrease in TICBT group as compared to brief treatment across post‐treatment, 6 and 12‐month	NR	**TICBT:** 22/92 = 24% **Brief treatment :** 4/88 = 5%
Steel et al. (2017)	Multicentre RCT (1b) *n* = 61	Schizophrenia, schizo‐affective disorder or schizophreniform disorder (DSM‐IV criteria); plus PTSD (DSM‐IV criteria); UK; 2 outpatient services	TICBT (psychoeducation, cognitive restructuring) Minimum: 6 Mean: 12.3 Maximum: 16 Length of time: NR	**LMM analysis (baseline to post‐treatment and 6 months):** **PANSS positive:** **Table:** Baseline mean Post‐treatment mean TICBT 19.1 17.8 TAU 18.3 19.8 Non statistically significant decrease in TICBT group as compared to TAU following treatment and 6‐month follow‐up **PANSS negative:** **Table:** Baseline mean Post‐treatment mean TICBT 16.3 15 TAU 15.3 16.4 Significant decrease in TICBT group as compared to TAU following treatment: *t* = 2.31 (*p* = 0.03), Hedges' *g* = 0.45. Non statistically significant decrease in TICBT group as compared to TAU at 6‐month follow‐up **PSYRATS‐AHRS:** **Table:** Baseline mean Post‐treatment mean TICBT 16.9 16.8 TAU 16.4 14 Non statistically significant increase in TICBT group as compared to TAU following treatment and 6‐month follow‐up **PSYRATS‐DRS:** **Table:** Baseline mean Post‐treatment mean TICBT 11.8 10 TAU 12.5 10.7 Non statistically significant increase in TICBT group as compared to TAU following treatment; non statistically significant decrease in TICBT group as compared to TAU at 6‐month follow‐up	**LMM analysis (pre to 6 and 12 months):** **CAPS‐S:** Non statistically significant increase in TICBT group as compared to TAU following treatment and 6‐month follow‐up **BDI:** Non statistically significant decrease in TICBT group as compared to TAU following treatment; non statistically significant increase in TICBT group as compared to TAU at 6‐month follow‐up B**AI:** Non statistically significant decrease in TICBT group as compared to TAU following treatment and 6‐month follow‐up **QLS:** Non statistically significant decrease in TICBT group as compared to TAU following treatment and 6‐month follow‐up	NR	**TICBT:** 4/27 = 15%
Trappler and Newville (2007)	Multicentre case series (4) *n* = 24	Diagnosis of schizophrenia or schizoaffective disorder (DSM‐IV criteria); plus PTSD (DSM‐IV criteria); USA; 3 inpatient services	Group TICBT (emotion regulation and behavior/coping strategies to trauma triggers) Minimum: NR Average: NR Maximum: 12 Length of time: 12 weeks	**Wilcoxon signed rank test (pre and post):** **BPRS total:** **Table:** Baseline mean Post‐treatment mean TICBT NR NR Sup. Psy. NR NR Significant decrease in TICBT group following treatment: *z* = −4.20 (*p* < 0.001), *r* = −0.9. Non statistically significant decrease in the supportive psychotherapy group **BPRS subscale—hallucinatory behaviour:** **Table:** Baseline mean Post‐treatment mean TICBT NR NR Sup. Psy. NR NR Non statistically significant decrease in TICBT group following treatment. Non statistically significant decrease in the supportive psychotherapy group **BPRS subscale—unusual thought content:** **Table:** Baseline mean Post‐treatment mean TICBT NR NR Sup. Psy. NR NR Significant decrease in TICBT group following treatment: *z* = −2 (*p* = 0.046), *r* = −0.43. Non statistically significant decrease in the supportive psychotherapy group **BPRS subscale—suspiciousness:** **Table:** Baseline mean Post‐treatment mean TICBT NR NR Sup. Psy. NR NR Significant decrease in TICBT group following treatment: *z* = −4.24 (*p* < 0.001), *r* = −0.9. Significant decrease in the supportive psychotherapy group following treatment: *z* = −2.07 (*p* = 0.039), *r* = −0.44	**Wilcoxon signed rank test (pre and post):** **IES:** Significant decrease in TICBT group following treatment: *z* = −3.47 (*p* = 0.001), *r* = −0.74. Non statistically significant decrease in the supportive psychotherapy group	NR	NR
TFCBT							
Keen et al. (2017)	Case series (4) *n* = 9	Schizophrenia spectrum disorder or PTSD or psychotic depression (ICD‐10 criteria); all reported psychotic symptoms (PANSS); UK; 1 outpatient service	TF‐CBTp (stabilization, cognitive restructuring, exposure (imagery rescripting or reliving), schema work) Minimum: 22 Median: 41 Maximum: 66 Length of time: 8–35 months	**Mean change:** **PSYRATS‐AHRS:** **Table:** Baseline mean Post‐treatment mean TFCBT 29.56 20.5 63% showed decrease following treatment, 29% showed reliable change (RCI) at 6‐month follow‐up **PSYRATS‐DRS:** **Table:** Baseline mean Post‐treatment mean TFCBT 13.57 8.33 67% showed decrease following treatment, 43% showed reliable change (RCI) at 6‐month follow‐up	**Mean change:** **PDS:** 80% showed decrease following treatment, 25% showed reliable change (RCI) at 6‐month follow‐up Mean at baseline: 37.22 Mean following treatment: 23.38 Mean at 6‐month follow‐up: 23.6 **BDI‐II/DASS:** 88% showed decrease following treatment, 40% showed reliable change (RCI at 6‐month follow‐up) BDI‐II mean at baseline: 34.5 BDI‐II mean following treatment: 24.9 BDI‐II mean at 6‐month follow‐up: 23 **BAI:** 63% showed decrease following treatment, 40% showed reliable change (RCI) at 6‐month follow‐up Mean at baseline: 32.33 Mean following treatment: 21.4 Mean at 6‐month follow‐up: 20.33	None: No adverse events or symptom exacerbation	0/9 = 0%
McCartney et al. (2019)	Case report (5) *n* = 1	First‐episode psychosis (meeting criteria for EIP service); UK; outpatient service	TFCBT (coping skills, imagery rescripting exposure) 22 sessions Length of time: NR	**PSYRATS‐AHRS:** **Table:** Baseline score Post‐treatment score TFCBT 36 23 Reliable but not clinically significant change following treatment	**IES:** Baseline: 70 Post‐intervention: 27 6‐month follow‐up: 27 Below level of probable PTSD following treatment **DASS:** Baseline: 40 Post‐intervention: 24 6‐month follow‐up: 31 No reliable or clinically significant change	An increase in voice frequency occurred at mid‐treatment, but this was not appraised negatively by the participant, with low distress. This resolved later in treatment	N/A
Callcott et al. (2004)	Case report (5) *n* = 1	Schizophrenia (ICD‐10 criteria); plus PTSD (ICD‐10 criteria); UK; outpatient service	TFCBT (exposure, imagery rescripting cognitive restructuring) 17 sessions Length of time: NR	**CPRS:** **Table:** Baseline score Post‐treatment score TFCBT 60 22 **SANS:** **Table:** Baseline Post‐treatment TFCBT 8 4	**IES:** Baseline: 41 Post‐intervention: 10	NR	N/A
Ward‐Brown et al. (2018)	Case report (5) *n* = 1	First‐episode psychosis (meeting criteria for EIP service); UK; outpatient service	TFCBT and EMDR (coping strategies, imagery rescripting, prolonged in‐vivo exposure, exposure via EMDR, reliving) 33 sessions Length of time: 1 year	**PSYRATS‐AHRS:** **Table:** Baseline score Post‐treatment score TFCBT 33 29	**IES‐R:** Baseline: 71 Post‐intervention: 25 6‐month follow‐up: 8 **CAPS:** Baseline: 87 Post‐intervention: 24 6‐month follow‐up: 4 **BDI‐II:** Baseline: 18 Post‐intervention: 17 6‐month follow‐up: 1 **BAI:** Baseline: 25 Post‐intervention: 13 6‐month follow‐up: 1 **WSAS:** Baseline: 19 Post‐intervention: 7 6‐month follow‐up: 0	NR	N/A
EMDR							
de Bont et al. (2016)	Multicentre RCT (1b) *n* = 155	Lifetime diagnosis of a psychotic disorder (MINI plus criteria) plus chronic PTSD (DSM‐IV‐TR criteria on the CAPS); Netherlands; 13 outpatient services	EMDR or PE Minimum: 8 Mean (PE): 7.1 Mean (EMDR): 7.8 Maximum: 8 Length of time: 8 weeks	**PE versus TAU** **LMM analysis** **PSYRATS‐AHRS:** **Table:** Baseline mean Post‐treatment mean PE 21.7 18.8 TAU 23 24.2 Non statistically significant decrease in PE group as compared to TAU following treatment and across all time points; non statistically significant increase in PE group as compared to TAU at 6‐month follow‐up **GPTS:** **Table:** Baseline mean Post‐treatment mean PE 88.8 67.3 TAU 83.8 82.7 Significant decrease in PE group as compared to TAU following treatment: *t* = −2.86 (*p* = 0.005), Hedges' *g* = 0.62 Significant decrease in PE group as compared to TAU at 6‐month follow‐up: *t* = −2.46. (*p* = 0.015), Hedges' *g* = 0.54 PE superior to TAU across all time points: *t* = −3.03 (*p* = 0.003) **No significant changes on GPTS between 6‐ and 12‐month follow‐up (van den Berg et al., 2018)** **GEE sensitivity analysis (odds ratio):** **Remission from psychotic disorders (SCI‐SR‐PANSS):** PE participants significantly more likely than TAU participants to be in remission following treatment: OR = 3.39 (*p* = 0.008) EMDR superior to TAU following treatment: OR = 3.17 (*p* = 0.013) PE participants non statistically significantly more likely than TAU participants to be in remission at 6‐month follow‐up PE superior to TAU across all time points: OR = 2.325 (*p* = 0.020) **No significant changes in the number of participants in remission at 12‐month follow‐up (van den Berg et al., 2018)**	**PE versus TAU** **LMM analysis** **BDI‐II:** Significant decrease in PE group as compared to TAU following treatment: t175 = −3.61 (*p* < 0.001); Hedges' *g* = 0.77 Significant decrease in PE group as compared to TAU at 6‐month follow‐up: t177 = −2.92 (*p* = 0.004), Hedges' *g* = 0.64 PE superior to TAU over time: *t* = −3.52 (*p* = 0.001) **No significant changes on BDI‐II between 6 and 12‐month follow‐up (van den Berg et al., 2018)** **PSP:** Non statistically significant increase in PE group as compared to TAU following treatment, 6‐month follow‐up, and across all time points **Paired sample** *t* **‐test:** **PSP:** Significant decrease between 6‐ and 12‐month follow‐up in PE group: *t* = 4.31, (*p* < 0.001)	None: Fewer participants in the trauma‐focused conditions experienced symptom exacerbation or adverse events as compared to the TAU condition	**EMDR:** 11/53 = 21% **PE:** 13/53 = 25% **No statistically significant difference in dropout between EMDR and PE** (**van den Berg et al., 2015)**
				**EMDR** versus **TAU** **LMM analysis (ITT)** **PSYRATS‐AHRS:** **Table:** Baseline mean Post‐treatment mean EMDR 24.5 16.8 TAU 23 24.2 Non statistically significant decrease in EMDR group as compared to TAU following treatment and 6‐month follow‐up. Non statistically significant increase in EMDR group as compared to TAU across all time points **GPTS:** **Table:** Baseline mean Post‐treatment mean EMDR 82.7 68 TAU 83.8 82.7 Significant decrease in EMDR group as compared to TAU following treatment: *t* = −2.68 (*p* = 0.008), Hedges' *g* = 0.57 Non statistically significant decrease in EMDR group as compared to TAU at 6‐month follow‐up EMDR superior to TAU across all time points: *t* = −2.38 (*p* = 0.019). **No significant changes on any measure between 6 and 12‐month follow‐up (van den Berg et al., 2018)** **GEE sensitivity analysis (odds ratio):** **Remission from psychotic disorders (SCI‐SR‐PANSS):** EMDR participants significantly more likely than TAU participants to be in remission following treatment: OR = 3.17 (*p* = 0.013) EMDR participants non statistically significantly more likely than TAU participants to be in remission at 6‐month follow‐up and across all time points **No significant changes in the number of participants in remission at 12‐month follow‐up (van den Berg et al., 2018)**	**EMDR** versus **TAU** **LMM analysis (ITT)** **BDI‐II:** Non statistically significant decrease in EMDR group as compared to TAU following treatment, 6‐month follow‐up and across all time points **No significant changes on BDI‐II between 6 and 12‐month follow‐up (van den Berg et al., 2018)** **PSP:** Non statistically significant increase in EMDR group as compared to TAU following treatment, 6‐month follow‐up and across all time points **Paired sample** *t* **‐test:** **PSP:** Significant decrease in EMDR group as compared to TAU between 6 and 12‐month follow‐up in EMDR: *t* = 2.08 (*p* = 0.044)		
Kim et al. (2010)	RCT (2b) *n* = 45	Diagnosis of schizophrenia (DSM‐IV criteria), inpatient stay of over 1 week; South Korea; 1 inpatient service	EMDR Minimum: 3 Average: NR Maximum: 3 Length of time: 3 weeks	**Repeated measures ANOVA:** **PANSS total:** **Table:** Baseline mean Post‐treatment mean EMDR 73.1 62.7 PMR 69.8 61.7 TAU 76.8 67.2 Non statistically significant decrease in EMDR group as compared to PMR and TAU **PANSS positive:** **Table:** Baseline mean Post‐treatment mean EMDR 16.9 12.2 PMR 15.9 12.9 TAU 18.8 15.4 Non statistically significant increase in EMDR group as compared to PMR; non statistically significant decrease in EMDR group as compared to TAU **PANSS negative:** **Table:** Baseline mean Post‐treatment mean EMDR 18.7 16.2 PMR 18.5 17.4 TAU 18.5 17.4 Non statistically significant decrease in EMDR group as compared to PMR and TAU	**Repeated measures ANOVA:** **HAM‐D:** Non statistically significant decrease in EMDR group as compared to PMR and TAU **HAM‐A:** Non statistically significant decrease in EMDR group as compared to PMR and TAU	None: No participant showed any exacerbation of symptoms and no participant had to withdraw due to a worsening of their condition	**Emdr:** 2/15 = 13% **PMR:** 1/15 = 7% **TAU:** 2/15 = 13% **No statistically significant difference in rate of dropout**
Slotema et al. (2019)	Case series (4) *n* = 47	Personality disorder (DSM‐IV‐TR criteria); plus PTSD (DSM‐IV‐TW criteria); Netherlands; 1 outpatient service	EMDR. Participants were undergoing other therapies simultaneously in TAU (psychodynamic psychotherapy (23%), CBT (2%), schema‐focused therapy (18%), DBT (7%), supporting sessions (39%), family therapy (7%) or other therapy (5%) Minimum: 2 Median: 4 Maximum: 15	**Wilcoxon signed rank test last observation carried forward (pre to post):** **PSYRATS‐AHRS (median):** **Table:** Baseline mean Post‐treatment mean EMDR 0 0 Significant decrease following treatment: *Z* = −2.12 (*p* = 0.034), Hedges' *g* = 0.2 **Without observation carried forward:** Non statistically significant decrease following treatment	*t* **‐test for dependent samples (pre to post):** **PDS:** Significant decrease following treatment: *t* = 7.94, (*p* < 0.001), Hedges' *g* = 2.26	EMDR treatment was completed by 68% of the sample EMDR treatment was experienced as stressful with an increase of instability in 4 participants, leading to dropout	15/47 = 32%
van den Berg and van der Gaag (2012)	Multicentre case series (4) *n* = 27	PTSD (criteria not reported); plus a lifetime schizophrenia spectrum disorder (criteria not reported); Netherlands; 4 outpatient services	EMDR, focused on trauma that caused current PTSD Minimum: NR Mean: 4.72 Maximum: 6 Length of time: 6 weeks	**Wilcoxon signed rank tests (pre to post):** **PSYRATS‐AHRS:** **Table:** Baseline mean Post‐treatment mean EMDR NR NR Significant decrease following treatment: *z* = −2.17 (*p* < 0.030), *r* = 0.33 **PSYRATS‐DRS:** **Table:** Baseline mean Post‐treatment mean EMDR NR NR Significant decrease following treatment: *z* = −2.02 (*p* < 0.043), *r* = 0.30 **PSYRATS total:** **Table:** Baseline mean Post‐treatment mean EMDR NR NR Significant decrease following treatment: *z* = −2.67 (*p* < 0.008), *r* = 0.40 **Paired sample** *t* **‐test:** **Table:** Baseline mean Post‐treatment mean EMDR 72.1 65.6 **GPTS:** Non statistically significant decrease following treatment	**Paired sample** *t* **‐test (pre to post):** **CAPS:** Significant decrease following treatment: *t* = 7.26 (*p* = 0.000), Hedges' *g* = 1.49 **PSS‐SR:** Significant decrease following treatment: *t* = 6.23 (*p* = 0.000), Hedges' *g* = 1.28 **BDI‐II:** Significant decrease following treatment: *t* = 4.81 (*p* = 0.000), Hedges' *g* = 0.99 **BAI:** Significant decrease following treatment: *t* = 4.4 (*p* = 0.000), Hedges' *g* = 0.91	3 participants reported increased stress or PTSD symptoms. In these cases, one session was dedicated to coping skills which was enough to help them regain control and motivation for treatment 2 participants contacted their case manager to discuss increased arousal, which later resolved 1 participant had a single relapse into drug use after leaving the home for the first time alone in years No other adverse events were reported	5/27 = 19%
de Bont et al. (2013)	Case series (4) *n* = 10	Under treatment for current psychotic symptoms; plus PTSD (DSM‐IV criteria); Netherlands, 1 outpatient service	PE or EMDR Minimum: NR Mean (PE): 9 Mean (EMDR): 11.5 Maximum: 12 Length of time: NR	**Wilcoxon pairwise test:** **PSYRATS‐AHRS (estimated marginal means):** **Table:** Baseline mean Post‐treatment mean EMDR 14.54 10.67 Non statistically significant decrease following treatment and 3‐month follow‐up **PSYRATS‐DRS (estimated marginal means):** **Table:** Baseline mean Post‐treatment mean EMDR 5.68 1.49 Non statistically significant decrease following treatment and 3‐month follow‐up	**Wilcoxon pairwise test:** **PSS‐SR:** Significant decrease following treatment: *r* = 0.73 (*p* < 0.001) and 3‐month follow‐up (*p* < 0.001) **CAPS:** Significant decrease following treatment: *Z* = −1.96 (*p* = 0.05), *r* = 0.49 and 3‐month follow‐up: *Z* = −2.52 (*p* = 0.012), *r* = 0.63 **OQ‐45.2:** Significant decrease following treatment: *Z* = −2.19 (*p* = 0.028), *r* = 0.69 and 3‐month follow‐up: *Z* = −2.37 (*p* = 0.018), *r* = 0.75 **SFS:** Non statistically significant increase following treatment and 3‐month follow‐up	None: No participants showed an increase in symptoms or deterioration in social functioning or clinically adverse events	**Emdr:** 1/5 = 20% **PE:** 1/5 = 20%
Yaşar et al. (2017)	Case report (5) *n* = 1	Schizophrenia (criteria not reported); Turkey; inpatient service	EMDR 2 sessions Length of time: 2 weeks	**PANSS:** **Table:** Baseline score 5‐month follow‐up score EMDR 78 34 **BPRS:** **Table:** Baseline score 5‐month follow‐up score EMDR 37 3	**CAPS:** Baseline: 96 5‐month follow‐up: 12 **IES‐R:** Baseline: 53 Post‐intervention: 25 5‐month follow‐up: 15 **BDI:** Baseline: 30 Post‐intervention: 16 5‐month follow‐up: 11 **CDSS:** Baseline: 16 5‐month follow‐up: 6 **BAI:** Baseline: 37 Post‐intervention: 24 5‐month follow‐up: 4	NR	N/A
Other							
Brand and Loewenstein (2013)	Case series (4) *n* = 237	DID (criteria not reported); 19 countries; many outpatient services	Phasic trauma treatment for DID Minimum: NR Average: NR Maximum: NR Length of time: NR	**ANOVA (across 6‐, 18‐ and 30‐month follow‐up):** **SCL‐90‐R—hearing voices item:** **Table:** Baseline mean 6‐month mean 1.89 1.63 Significant decrease across all time points: *F* = 3.40 (*p* = 0.02) Hedges' *g* at 6‐month of treatment: 0.16 Hedges' *g* at 18‐month of treatment: 0.25 Hedges' *g* at 30‐month of treatment: 0.32	N/A	NR	NR
Paulik et al. (2019)	Case series (4) *n* = 12	Currently hearing voices; plus experiencing PTSD symptoms that appear directly or indirectly linked to the voices (no symptom threshold); Australia; 1 outpatient service	Imagery rescripting (exposure) Minimum: NR Average: NR Maximum: 10 Length of time: 9–19 weeks	**LMM analysis over time (baseline, mid‐intervention, post‐treatment):** **PSYRATS‐AHRS distress:** **Table:** Baseline mean Post‐treatment mean 16 12 Significant decrease across all time points: *t* = −3.33 (*p* = 0.01) Hedges' *g* following treatment: 0.69 No significant increase from post‐treatment to 3‐month follow‐up **PSYRATS‐AHRS frequency:** **Table:** Baseline mean Post‐treatment mean 9 6 Significant decrease across all time points: *t* = −7.47 (*p* < 0.001), Hedges' *g* = 0.74 No significant increase from post‐treatment to 3‐month follow‐up **BAVQ (malevolence):** **Table:** Baseline mean Post‐treatment mean 9 8 Significant decrease across all time points: *t* = 2.22 (*p* = 0.033), Hedges' *g* = 0.34 **BAVQ (omnipotence):** **Table:** Baseline mean Post‐treatment mean 11 10 Non statistically significant decrease across all time points	**LMM analysis over time (baseline, mid‐intervention, post‐treatment):** **PSS:** Significant decrease over time: *t* = −3.62 (*p* = 0.005), Hedges' *g* = 0.74 **DASS depression:** Non statistically significant decrease across all time points **DASS anxiety:** Non statistically significant decrease across all time points	Two patients reported an initial increase in intrusions which lasted 1 week. No other adverse events occurred	1/12 = 8%
Strous et al. (2005)	Multicentre case series (4) *n* = 24	Schizophrenia diagnosis, holocaust survivors; Israel; 2 inpatient services	3‐h video testimony session over 1–2 sessions	**Paired** *t* **‐test (baseline and post):** **PANNS total:** **Table:** Baseline mean Post‐treatment mean 68.6 69.8 Non statistically significant increase following treatment **PANNS positive:** **Table:** Baseline mean Post‐treatment mean 14.4 14.3 Non statistically significant decrease following treatment **PANNS negative:** **Table:** Baseline mean Post‐treatment mean 22.9 23.9 Non statistically significant increase following treatment **PANNS global:** **Table:** Baseline mean Post‐treatment mean 31.4 31.6 Non statistically significant increase following treatment	**Paired** *t* **‐test (baseline and post):** **CAPS:** Significant decrease following treatment: *t* = 4.2, (*p* < 0.001) Hedges' *g* = 0.65	None: No short‐term or long‐lasting adverse events were reported	0/24 = 0%
Arens (2014)	Case report (5) *n* = 1	Combat PTSD and hallucinations; USA; 1 outpatient service	Trauma management therapy (15 VR assisted imaginal and in‐vivo exposure with 14 group social and emotional skill sessions) Length of time: 3 weeks	**No. auditory hallucinations per week:** **Table:** Baseline Post‐treatment 7 1 **No. visual hallucinations per week:** **Table:** Baseline Post‐treatment 2 2	**CAPS:** Baseline: 91 Post‐intervention: 21 3‐month follow‐up: 33 **PCL‐M:** Baseline: 64 Post‐intervention: 28 3‐month follow‐up: 23 **Global anxiety (0–10):** Baseline: 6.4 Post‐intervention: 1.3 3‐month follow‐up: 1.1 **No. social activities per week:** Baseline: 0 Post‐intervention: 5 3‐month follow‐up: 0	A slight increase in hallucinations in the first week of treatment, which resolved in the following weeks	N/A

Abbreviations: BAI, Beck Anxiety Inventory; BAVQ, Beliefs About Voices Questionnaire; BDI, Beck Depression Inventory; BDI‐II, Beck Depression Inventory‐II; BDI‐II/DASS, Depression Anxiety Stress Scales; BPRS, Brief Psychiatric Rating Scale; CAPS, Clinician Administered PTSD Scale; CAPS‐S, Clinician‐Administered PTSD Scale for Schizophrenia; CDSS, Calgary Depression Scale for Schizophrenia; CPRS, Comprehensive Psychopathological Rating Scale; DID, dissociative identity disorder; GPTS, Green et al. Paranoid Thoughts Scale; HAM‐A, Hamilton Anxiety Rating Scale; HAM‐D, Hamilton Depression Rating Scale; IES, Impact of Events Scale; IES‐R, Impact of Events Scale‐Revised; NR, not reported; OQ‐45.2, Outcome Questionnaire‐45.2; PANSS, Positive and Negative Syndrome Scale; PCL‐M, PTSD Checklist—Military version; PDS, Posttraumatic Diagnostic Scale; PE, prolonged exposure; PSP, Personal and Social Performance scale; PSS, Perceived Stress Scale; PSS‐SR, PTSD Symptom Scale**—**Self‐Report; PSYRATS‐AHRS, Psychotic Symptom Rating Scale**—**Auditory Hallucinations Rating Scale; PSYRATS‐DRS, Delusions Rating Scale; QLS, Quality of Life Scale; RCT, randomised controlled trial; SANS, Scale for the Assessment of Negative Symptoms; SCI‐SR‐PANSS, Structured Clinical Interview for Symptoms of Remission for the PANSS; SCL‐90‐R, Symptom Checklist‐90‐Revised; SFS, Social Functioning Scale; WSAS, Work and Social Adjustment Scale.

Risk of bias is summarised in Tables S1–S3. Each RCT held ‘some concerns’ regarding risk of bias. The case series were mostly of acceptable risk of bias, though two were assessed to be of a higher risk of bias (Brand & Loewenstein, 2013; Trappler & Newville, 2007). The case reports were all assessed to meet an appropriate quality level for inclusion.

### 3.1 Trauma‐informed CBT

Two of three TICBT studies (Mueser et al., 2015; Steel et al., 2016) were RCTs following the same protocol teaching cognitive restructuring to challenge trauma‐related thoughts and beliefs, meaning no exposure techniques were used (Ehlers & Clark, 2008). TICBT was not superior to the respective control groups in reducing positive psychotic symptoms. The third study (Trappler & Newville, 2007) was a case series using Cloitre's Skill Training in Affect Regulation preparatory work (Cloitre et al., 2002). It found significant decreases in measures of overall psychotic symptoms, delusions, and paranoia. Only paranoia also significantly decreased in a matched group undergoing supportive psychotherapy.

Regarding secondary outcomes, one of the two controlled studies (Mueser et al., 2015) found a small but statistically significant decrease in PTSD symptoms as compared to control. The non‐controlled study (Trappler & Newville, 2007) found a significant effect for its treatment programme on PTSD symptoms, with no effect found in its matched supportive psychotherapy group. There were no significant effects in these studies on measures of depression, anxiety nor quality of life.

### 3.2 Trauma‐focused CBT

The TFCBT studies utilised a variety of exposure techniques, such as imagery rescripting and reliving. To qualify as TFCBT, it is necessary that therapeutic techniques intended to process and modify unhelpful peri‐ or post‐traumatic thoughts and feelings, such as cognitive distortions, guilt, and shame, are included. Each included TFCBT study was non‐controlled. Due to lack of comprehensive raw data and statistical analysis conducted by the authors, effect sizes were not able to be calculated for these studies. One (Keen et al., 2017) found decreases in auditory hallucinations and delusions scores, 29% and 43% respectively of which remained clinically significant at 6‐month follow‐up. Of the case reports, two (Callcott et al., 2004; Ward‐Brown et al., 2018) found decreases in their measures of psychotic symptoms. The final included case report (McCartney et al., 2019) found a reliable but not clinically significant decrease in auditory hallucinations.

Regarding secondary outcomes, PTSD symptoms decreased in all TFCBT studies. Consistent decreases in depression and anxiety measures were also found. Only one study (Ward‐Brown et al., 2018) included a quality‐of‐life scale, reporting a substantial improvement.

### 3.3 Eye movement desensitisation and reprocessing

All the EMDR studies included in this review used the standard eight‐phase protocol (Shapiro, 2001). This protocol focuses mostly on exposure, although one phase focuses on ‘installing’ more helpful thoughts and cognitions. Whilst EMDR had the greatest number of included studies, the evidence of effectiveness was mixed. One RCT (de Bont et al., 2016) found EMDR was superior to the control in reducing paranoia following treatment as well as across all time points. EMDR was also associated with greater likelihood of remission from a psychotic disorder following treatment, but this was no longer statistically significant at 6‐month follow‐up. This contrasts with the other included RCT (Kim et al., 2010), which found no statistically significant differences on psychotic symptoms between EMDR and either of its two control conditions. The case series and case reports provide mixed evidence of small effects on specific symptom domains, particularly hallucinations (Slotema et al., 2019; van den Berg & van der Gaag, 2012) and overall symptoms (van den Berg & van der Gaag, 2012; Yasar et al., 2017). No study found a specific effect on paranoia.

Regarding secondary outcomes, no controlled study reported on PTSD symptoms. Four studies found large decreases on PTSD symptom measures (de Bont et al., 2013; Slotema et al., 2019; van den Berg & van der Gaag, 2012; Yasar et al., 2017). Regarding depression, the two controlled studies found no superior effect for EMDR over the control following treatment (de Bont et al., 2016; Kim et al., 2010). Uncontrolled studies found decreases in depression measures (van den Berg & van der Gaag, 2012; Yasar et al., 2017). One controlled study measured anxiety finding no effect for EMDR over the controls (Kim et al., 2010). Two uncontrolled studies reported decreases in anxiety measures, reaching significance when significance testing was possible (van den Berg & van der Gaag, 2012). For quality‐of‐life measures, one controlled study found no superior effect for EMDR over control following intervention or 6‐month follow‐up (de Bont et al., 2016), whilst one uncontrolled study found a significant improvement following treatment and 3‐month follow‐up (de Bont et al., 2013).

### 3.4 Prolonged Exposure

Two studies used PE (Foa et al., 2007), a protocol which focuses solely on exposure without a cognitive component. One RCT (de Bont et al., 2016) found a superior effect for PE over control in reducing paranoia following treatment, which sustained at 6 and 12‐month follow‐up (van den Berg et al., 2018). In addition, PE participants were significantly more likely to be in remission from a psychotic disorder at follow‐up. No significant impact for PE over the control for auditory hallucinations was found. A study that combined participants that had undergone PE or EMDR as one treatment group has been reported above (de Bont et al., 2013).

Regarding secondary outcomes, PE resulted in statistically significant decreases on depression scores following treatment and 6‐month follow‐up compared to the control, but not on social functioning scores (de Bont et al., 2016).

### 3.5 Other interventions

One case series investigated the effects of ‘phasic trauma treatment’ for Dissociative Identity Disorder (Brand & Loewenstein, 2013) (incorporating an exposure component), described in the Guidelines for Treating Dissociative Identity Disorder in Adults (International Society for the Study of Trauma and Dissociation, 2011). They found this treatment had a significant impact on a hearing voices item, with effect sizes growing larger the longer the person was in treatment.

There were no secondary outcomes reported relevant to this review within this study.

One case series investigated the effects of imagery rescripting (Paulik et al., 2019). This intervention had a beneficial impact on voice hearing, with auditory hallucination distress and frequency, and belief in voice malevolence seeing significant decreases following treatment, sustained at 3‐month follow‐up. However, belief in voice omnipotence did not significantly decrease following treatment.

This study reported on PTSD symptomology, finding a significant decrease following treatment. No significant effect was found on depression or anxiety measures (Paulik et al., 2019).

One case series investigated the effects of a one‐off video interview regarding a personal experience of the Holocaust, with a focus on details about losses and grief experienced (Strous et al., 2005). There was no effect on psychotic nor PTSD symptoms.

One case report (Arens, 2014) looked at the effectiveness of trauma management therapy (Turner et al., 2005) (TMT) in a combat veteran. TMT incorporates in vivo and virtual reality assisted imaginal exposure sessions together with group social and emotional coping skills. The number of self‐reported auditory and visual hallucinations declined from 7 to 1, and 2 to 0 respectively by 3‐month follow‐up. Two measures of PTSD symptomology showed a decrease at 3‐month follow‐up. Anxiety scores had also decreased at 3‐month follow‐up.

### 3.6 Tolerability and acceptability

Regarding tolerability, 41% (7/17) of all studies and 100% (3/3) of the non‐exposure based protocols did not report on adverse events or harm. Of the exposure‐based studies, 29% (4/14) did not report on harm, 36% (5/14) reported that there were no instances of adverse events or harm, with one (de Bont et al., 2016) reporting that fewer participants in the active condition (EMDR) experienced symptom exacerbation or adverse events than in the control condition. 29% (4/14) reported a brief symptom exacerbation early in treatment that resolved later, sometimes with brief (i.e. one session or one conversation) additional support provided (e.g. psychoeducation about increased arousal when starting a new intervention). Only one reported exacerbation that may not have resolved; in this case stress and an increase in instability leading to dropouts in 4/47 (9%) of their participants, with only 32/47 (68%) completing EMDR treatment (Slotema et al., 2019).

Regarding acceptability, 17% (2/12) of studies did not report on dropouts (five studies have not been included in this calculation due to being case reports). We amalgamated dropout rates within treatment modality (excluding again case reports) and found a dropout rate of 22% (26/119 participants) within TICBT studies, 0% (0/9) within TFCBT studies, 23% (34/147) within EMDR studies, 24% (14/58) within PE studies and 3% for other interventions (1/36 participants). In total, this results in a dropout rate of 22% (26/119 participants) for non exposure‐based protocols and 20% (49/250 participants) for exposure‐based protocols.

## 4 DISCUSSION

In our systematic review with broad inclusion criteria, we found only 17 studies of TFPT in psychosis, with the literature largely comprised of case series. Nevertheless, several well‐controlled studies indicate that TICBT, which does not utilise exposure, is not effective at reducing psychotic symptoms. Regarding those approaches that do use exposure to facilitate trauma memory reprocessing, the majority of the high‐quality evidence results from the large multicentre RCT of de Bont et al. (2016). Though this study provided evidence in favour of PE and EMDR, the study protocol included psychotic symptoms as a secondary outcome. Whilst included case studies and case reports further support the idea that exposure‐based interventions may be effective, the low robustness of the evidence base means that the effectiveness of TFPT for psychotic symptoms is equivocal.

Our finding of greater evidence for the use of exposure as compared to non exposure‐based interventions in reducing psychotic symptoms is in keeping with a previous review (Brand et al., 2018). In our review, 11 of the 14 studies utilising exposure found a positive impact for TFPT on at least one psychotic symptom, whilst one of three of the studies not utilising exposure did. This provides support for the view that the inclusion of trauma memory reprocessing is necessary to address psychotic symptoms in the context of trauma. This is also in‐keeping with a process analysis of intervention sessions which concluded that exposure is required to treat trauma symptoms in patients with psychosis (Steel et al., 2016).

A crucial point to consider regarding the studies in this review is that they were primarily oriented towards treating PTSD. This means the interventions were often not targeted to traumatic memories that may be directly or indirectly related to psychotic symptoms. This is important considering research which has shown relationships between the content of psychotic symptoms in trauma survivors and experiences of trauma (Hardy et al., 2005; McCarthy‐Jones et al., 2012; Onyeama et al., 2011; Peach et al., 2020; Vila‐Badia et al., 2021).

Several included studies which did show a positive effect of TFPT on psychotic symptoms described clear links between trauma and psychotic symptoms in their participants (Arens, 2014; Yasar et al., 2017). Paulik et al. (2019) also chose to focus their trauma intervention on traumas that appeared related to psychotic phenomena, and reported the largest effect sizes in the review (*g* = 0.69 for distress and *g* = 0.74 for frequency of voices following treatment), maintained at 3 months. Across all participants, trauma memory intrusions and voices reduced concurrently, suggesting the two symptom domains may be related by common underlying processes. This accords with theories suggesting that similar mechanisms are involved in PTSD and trauma‐induced psychotic symptoms (Bloomfield et al., 2021; Morrison et al., 2004) and that, in the context of trauma, auditory hallucinations may be a type of post‐traumatic intrusion related to a memory (Steel, 2015). Therefore, when a patient is able to construct a complete memory of the traumatic event through exposure during TFPT, the memory may stop being retrieved involuntarily through intrusions such as voices.

It may therefore be important psychotherapeutically to differentiate between focussing on a trauma memory that pre‐dates psychosis (in which case the trauma may be relevant to the aetiology of the psychosis) and a trauma memory that took place after the onset of psychosis. In the present review, many of the included studies included substantial proportions of post‐psychosis onset trauma (e.g. 18% (Steel et al., 2016), 18% (de Bont et al., 2016), 30% (van den Berg & van der Gaag, 2012)) which may have contributed to some of the null results. Future research will benefit from directly addressing this question.

The use of exposure for people with psychotic symptoms has been a concern for some clinicians, who believe that exposure may cause harm by exacerbating psychotic symptoms (Cragin et al., 2017). In this review, though some studies did report a temporary increase in distress and/or symptoms, this is a typical response to exposure in trauma treatment as it is designed to elicit and facilitate the therapeutic processing of distress (Foa et al., 2002). Overall, the majority of those that reported on harm reported no harm, with one study reporting fewer adverse events in the TFPT group than the control (de Bont et al., 2016). Furthermore, there was a similar dropout rate between non exposure‐based (22%; 26/119 participants) and exposure‐based (20%; 49/250 participants) protocols. These dropout rates are comparable to those of CBTp (Lincoln et al., 2008) (16%). This indicates that TFPTs, and exposure specifically, have acceptable levels of tolerability and acceptability. This finding accords with van den Berg et al. (2015) who reviewed adverse events during PE or EMDR with people with psychosis, finding that these treatments were associated with significantly less symptom exacerbation and adverse events than waitlist conditions.

It is important to consider the limitations of the existent literature. There were only four controlled studies included in this review, only two of which used an intervention meeting the criteria of TFPT (Ehlers & Clark, 2000). The methodological quality of studies was impacted by low sample sizes, lack of blinding and other methodological issues. As many studies did not specifically target psychotic symptoms, the inclusion criteria of studies did not always necessitate the high levels of psychotic symptoms in all participants, or for psychotic symptoms to be present at all (e.g. de Bont et al., 2013; Slotema et al., 2019). This means the studies may have had low power to detect and may fall victim to floor effects.

Our review has a number of strengths. We pre‐registered our review and adhered to the robust a priori criteria. We took a broad view of psychotic symptoms and did not limit inclusion criteria to any diagnosis, syndrome, or trauma type. We calculated Hedges' *g* where possible (a more reliable measure of effect size for small sample sizes) (Cumming, 2012). The broad range of studies included in the review and the use of risk of bias measures appropriate to each also represents a strength.

A limitation of our review is that it has not been possible to meta‐analyse the available data as we did not meet our a priori criteria due to the lack of controlled studies and variation in psychotherapeutic intervention. This has also rendered it not possible to differentiate further between types of TFPT that use exposure, for example, those that include a significant cognitive component compared to those that do not. There remains a risk of selection bias in this review as some forms of therapy would likely involve reappraisal of a trauma memory (e.g. learning that a traumatic experience was not their fault) and so could be described as trauma‐focused according to our broad criteria, but would not necessarily have been picked up our search.

Several clinical recommendations arise from this review. Given the evidence of a relationship between trauma and psychosis, there is a clear need for clinicians to consider and assess for histories of trauma and PTSD in people with psychotic symptoms (strong recommendation).

TFPT with exposure should not be withheld by default from all patients for fears of safety or causing harm. Consistent with previous reviews (Sin & Spain, 2016; Swan et al., 2017), we found that TFPT is effective in reducing PTSD symptoms in people with psychosis, and we also found that TFPT may reduce psychotic symptoms. Given that we further found TFPT to be well tolerated and acceptable, there appears to be little justification for withholding TFPT from people with psychosis (conditional recommendation).

Several research recommendations arise from this review. Most pressingly, more high‐quality research is needed, specifically with psychotic symptoms as a primary outcome and on patients with trauma histories. We look forward to the findings of controlled research which is underway (Peters, 2020; Valiente‐Gómez et al., 2020). Future research will benefit from a standardisation of the measure used for psychotic symptoms to enable direct comparison between studies; there have been recent (albeit controversial) movements to do so for non‐psychotic measures (The Lancet Psychiatry, 2020). The diversity of measures currently used in the literature serves as a barrier to understanding, obfuscating potentially promising findings. Furthermore, future research can compare outcomes for different types of TFPT that do utilise exposure. For example, TFCBT, which follows exposure with cognitive techniques to restructure and reappraise peri‐ and post‐traumatic cognitions, may differ from PE, which does not. Finally, future research can consider the question of whether targeting the memory reprocessing intervention to traumas that appear related to the psychotic symptoms is most effective. Such a study could also investigate if there is a difference in efficacy between people that identify a direct (e.g. hallucination as a trauma memory), indirect (content is thematically linked) or no association between the trauma and the psychotic symptoms. Researchers such as Peach et al. (2020) and Hardy et al. (2005) have suggested that direct associations indicate a key role of post‐traumatic intrusions, whilst indirect associations suggest a larger role of beliefs and emotions. This therefore indicates different treatment protocols may be more effective depending on the type of links that can be made between trauma and psychotic symptoms.

## 5 CONCLUSION

The evidence base of effectiveness for TFPT in traumatised people for reducing psychotic symptoms is currently weak. There is tentative evidence in favour of exposure‐based interventions which provides support for the view that the inclusion of trauma memory reprocessing may be necessary to treat psychotic symptoms in trauma survivors. Further well‐controlled studies of TFPT for psychotic symptoms are needed. Our analysis indicated that TFPT was well tolerated, and acceptability levels were comparable to other psychological interventions for psychotic symptoms. Therefore, there is little to suggest that TFPT for PTSD should be withheld from people with psychotic symptoms.

## AUTHOR CONTRIBUTIONS

**Jordan Reid**: Conceptualization; data curation; formal analysis; investigation; methodology; project administration; supervision; writing – original draft; writing – review & editing. **Charles Cole**: Data curation; formal analysis; investigation; validation. **Nabeela Malik**: Data curation; formal analysis; investigation; validation. **Vaughan Bell**: Conceptualization; methodology. **Michael Bloomfield**: Conceptualization; funding acquisition; methodology; supervision.

## CONFLICT OF INTEREST STATEMENT

The authors have declared that there are no conflicts of interest in relation to the subject of this study.

## Supporting information

Supporting Information S1

Tables S1–S3

## Data Availability

The authors confirm that the data supporting the findings of this study are available within the article and its supplementary materials.

## References

[mpr2005-bib-0001] Arens, A. (2014). Trauma management therapy for a veteran with co‐occurring combat PTSD and hallucinations. Clinical Case Studies, 14(2), 115–128. 10.1177/1534650114541324

[mpr2005-bib-0002] Bloomfield, M. , Chang, T. , Woodl, M. , Lyons, L. M. , Cheng, Z. , Bauer‐Staeb, C. , Hobbs, C. , Bracke, S. , Kennerley, H. , Isham, L. , Brewin, C. , Billings, J. , Greene, T. , & Lewis, G. (2021). Psychological processes mediating the association between developmental trauma and specific psychotic symptoms in adults: A systematic review and meta‐analysis. World Psychiatry, 20(1), 107–123. 10.1002/wps.20841 33432756 PMC7801841

[mpr2005-bib-0003] Brand, B. , & Loewenstein, R. (2013). Does phasic trauma treatment make patients with dissociative identity disorder treatment more dissociative? Journal of Trauma & Dissociation, 15(1), 52–65. 10.1080/15299732.2013.828150 24377972

[mpr2005-bib-0004] Brand, R. , McEnery, C. , Rossell, S. , Bendall, S. , & Thomas, N. (2018). Do trauma‐focussed psychological interventions have an effect on psychotic symptoms? A systematic review and meta‐analysis. Schizophrenia Research, 195, 13–22. 10.1016/j.schres.2017.08.037 28844432

[mpr2005-bib-0005] Buckley, P. , Miller, B. , Lehrer, D. , & Castle, D. (2008). Psychiatric comorbidities and schizophrenia. Schizophrenia Bulletin, 35(2), 383–402. 10.1093/schbul/sbn135 19011234 PMC2659306

[mpr2005-bib-0006] Callcott, P. , Standart, S. , & Turkington, D. (2004). Trauma within psychosis: Using a CBT model for PTSD in psychosis. Behavioural and Cognitive Psychotherapy, 32(2), 239–244. 10.1017/s1352465804001249

[mpr2005-bib-0007] Campbell, M. , McKenzie, J. , Sowden, A. , Katikireddi, S. V. , Brennan, S. E. , Ellis, S. , Hartmann‐Boyce, J. , Ryan, R. , Shepperd, S. , Thomas, J. , Welch, V. , & Thomson, H. (2020). Synthesis without meta‐analysis (SWiM) in systematic reviews: Reporting guideline. BMJ, l6890. 10.1136/bmj.l6890 31948937 PMC7190266

[mpr2005-bib-0008] Cloitre, M. , Koenen, K. , Cohen, L. , & Han, H. (2002). Skills training in affective and interpersonal regulation followed by exposure: A phase‐based treatment for PTSD related to childhood abuse. Journal of Consulting and Clinical Psychology, 70(5), 1067–1074. 10.1037/0022-006x.70.5.1067 12362957

[mpr2005-bib-0009] Cragin, C. , Straus, M. , Blacker, D. , Tully, L. , & Niendam, T. (2017). Early psychosis and trauma‐related disorders: Clinical practice guidelines and future directions. Frontiers in Psychiatry, 8, 33. 10.3389/fpsyt.2017.00033 28321193 PMC5337515

[mpr2005-bib-0010] Croft, J. , Heron, J. , Teufel, C. , Cannon, M. , Wolke, D. , Thompson, A. , Houtepen, L. , & Zammit, S. (2019). Association of trauma type, age of exposure, and frequency in childhood and adolescence with psychotic experiences in early adulthood. JAMA Psychiatry, 76(1), 79. 10.1001/jamapsychiatry.2018.3155 30477014 PMC6490231

[mpr2005-bib-0011] Cumming, G. (2012). Understanding the new statistics: Effect sizes, confidence intervals, and meta‐analysis. Routledge.

[mpr2005-bib-0012] de Bont, P. , van den Berg, D. , van der Vleugel, B. , de Roos, C. , de Jongh, A. , van der Gaag, M. , & van Minnen, A. M. (2016). Prolonged exposure and EMDR for PTSD v. a PTSD waiting‐list condition: Effects on symptoms of psychosis, depression and social functioning in patients with chronic psychotic disorders. Psychological Medicine, 46(11), 2411–2421. 10.1017/s0033291716001094 27297048

[mpr2005-bib-0013] de Bont, P. , van Minnen, A. , & de Jongh, A. (2013). Treating PTSD in patients with psychosis: A within‐group controlled feasibility study examining the efficacy and safety of evidence‐based PE and EMDR protocols. Behavior Therapy, 44(4), 717–730. 10.1016/j.beth.2013.07.002 24094795

[mpr2005-bib-0014] Ehlers, A. , & Clark, D. (2000). A cognitive model of posttraumatic stress disorder. Behaviour Research and Therapy, 38(4), 319–345. 10.1016/s0005-7967(99)00123-0 10761279

[mpr2005-bib-0015] Ehlers, A. , & Clark, D. (2008). Post‐traumatic stress disorder: The development of effective psychological treatments. Nordic Journal of Psychiatry, 62(sup47), 11–18. 10.1080/08039480802315608 18752113 PMC3059487

[mpr2005-bib-0016] Foa, E. , Hembree, E. , & Rothbaum, B. (2007). Prolonged exposure therapy for PTSD: Emotional processing of traumatic experiences, therapist guide. Oxford University Press.

[mpr2005-bib-0017] Foa, E. , Zoellner, L. , Feeny, N. , Hembree, E. , & Alvarez‐Conrad, J. (2002). Does imaginal exposure exacerbate PTSD symptoms? Journal of Consulting and Clinical Psychology, 70(4), 1022–1028. 10.1037/0022-006x.70.4.1022 12182265

[mpr2005-bib-0018] Foote, B. , & Park, J. (2008). Dissociative identity disorder and schizophrenia: Differential diagnosis and theoretical issues. Current Psychiatry Reports, 10(3), 217–222. 10.1007/s11920-008-0036-z 18652789

[mpr2005-bib-0019] Guyatt, G. , Oxman, A. , Vist, G. , Kunz, R. , Falck‐Ytter, Y. , Alonso‐Coello, P. , & Schünemann, H. J. (2008). GRADE: An emerging consensus on rating quality of evidence and strength of recommendations. BMJ, 336(7650), 924–926. 10.1136/bmj.39489.470347.ad 18436948 PMC2335261

[mpr2005-bib-0020] Hannerz, H. , Borgå, P. , & Borritz, M. (2001). Life expectancies for individuals with psychiatric diagnoses. Public Health, 115(5), 328–337. 10.1016/s0033-3506(01)00471-1 11593442

[mpr2005-bib-0021] Hardy, A. , Fowler, D. , Freeman, D. , Smith, B. , Steel, C. , Evans, J. , Garety, P. , Kuipers, E. , Bebbington, P. , & Dunn, G. (2005). Trauma and hallucinatory experience in psychosis. The Journal of Nervous and Mental Disease, 193(8), 501–507. 10.1097/01.nmd.0000172480.56308.21 16082293

[mpr2005-bib-0022] Hill, A. (1965). The environment and disease: Association or causation? Proceedings of the Royal Society of Medicine, 58(5), 295–300. 10.1177/003591576505800503 14283879 PMC1898525

[mpr2005-bib-0023] Howes, O. , & Murray, R. (2014). Schizophrenia: An integrated sociodevelopmental‐cognitive model. The Lancet, 383(9929), 1677–1687. 10.1016/s0140-6736(13)62036-x PMC4127444 24315522

[mpr2005-bib-0024] Institute of Health Economics . (2014). Quality appraisal of case series studies checklist. Ihe.ca. Retrieved November 30, 2021, from http://www.ihe.ca/research‐programs/rmd/cssqac/cssqac‐about

[mpr2005-bib-0025] International Society for the Study of Trauma and Dissociation . (2011). Guidelines for treating dissociative identity disorder in adults, third revision. Journal of Trauma & Dissociation, 12(2), 115–187. 10.1080/15299732.2011.537247 21391103

[mpr2005-bib-0026] Jablensky, A. (2010). The diagnostic concept of schizophrenia: Its history, evolution, and future prospects. Schizophrenia, 12(3), 271–287. 10.31887/dcns.2010.12.3/ajablensky PMC3181977 20954425

[mpr2005-bib-0027] Keen, N. , Hunter, E. , & Peters, E. (2017). Integrated trauma‐focused cognitive‐behavioural therapy for post‐traumatic stress and psychotic symptoms: A case‐series study using imaginal reprocessing strategies. Frontiers in Psychiatry, 8, 92. 10.3389/fpsyt.2017.00092 28620323 PMC5451497

[mpr2005-bib-0028] Kelleher, I. , Keeley, H. , Corcoran, P. , Ramsay, H. , Wasserman, C. , Carli, V. , Sarchiapone, M. , Hoven, C. , Wasserman, D. , & Cannon, M. (2013). Childhood trauma and psychosis in a prospective cohort study: Cause, effect, and directionality. American Journal of Psychiatry, 170(7), 734–741. 10.1176/appi.ajp.2012.12091169 23599019

[mpr2005-bib-0029] Kim, D. , Choi, J. , Kim, S. , Oh, D. , Park, S. , & Lee, S. (2010). A pilot study of brief eye movement desensitization and reprocessing (EMDR) for treatment of acute phase schizophrenia. Korean Journal of Biological Psychiatry, 17(2), 94–102.

[mpr2005-bib-0030] Lincoln, T. , Suttner, C. , & Nestoriuc, Y. (2008). Effects of cognitive interventions for schizophrenia: A meta‐analysis. Psychologische Rundschau, 59(4), 217–232. 10.1026/0033-3042.59.4.217

[mpr2005-bib-0031] McCarthy‐Jones, S. , Trauer, T. , Mackinnon, A. , Sims, E. , Thomas, N. , & Copolov, D. (2012). A new phenomenological survey of auditory hallucinations: Evidence for subtypes and implications for theory and practice. Schizophrenia Bulletin, 40(1), 231–235. 10.1093/schbul/sbs156 23267192 PMC3885292

[mpr2005-bib-0032] McCartney, L. , Douglas, M. , Varese, F. , Turkington, D. , Morrison, A. , & Dudley, R. (2019). Cognitive behavioural therapy for psychosis targeting trauma, voices and dissociation: A case report. The Cognitive Behaviour Therapist, 12, e18. 10.1017/s1754470x19000035

[mpr2005-bib-0033] Moher, D. , Liberati, A. , Tetzlaff, J. , & Altman, D. (2009). Preferred reporting items for systematic reviews and meta‐analyses: The PRISMA statement. PLoS Medicine, 6(7), e1000097. 10.1371/journal.pmed.1000097 19621072 PMC2707599

[mpr2005-bib-0034] Moola, S. , Munn, Z. , & Tufanaru, C. (2017). JBI systematic reviews checklist for case reports. Jbi.global. Retrieved November 30, 2021, from https://jbi.global/sites/default/files/2019‐05/JBI_Critical_Appraisal‐Checklist_for_Case_Reports2017_0.pdf

[mpr2005-bib-0035] Morrison, A. , Frame, L. , & Larkin, W. (2003). Relationships between trauma and psychosis: A review and integration. British Journal of Clinical Psychology, 42(4), 331–353. 10.1348/014466503322528892 14633411

[mpr2005-bib-0036] Morrison, A. , Renton, J. , Dunn, H. , Williams, S. , & Bentall, R. (2004). Cognitive therapy for psychosis: A formulation‐based approach. Routledge.

[mpr2005-bib-0037] Mueser, K. , Gottlieb, J. , Xie, H. , Lu, W. , Yanos, P. T. , Rosenberg, S. D. , Silverstein, S. M. , Duva, S. M. , Minsky, S. , Wolfe, R. S. , & McHugo, G. J. (2015). Evaluation of cognitive restructuring for post‐traumatic stress disorder in people with severe mental illness. British Journal of Psychiatry, 206(6), 501–508. 10.1192/bjp.bp.114.147926 PMC4450219 25858178

[mpr2005-bib-0038] Mueser, K. , Lu, W. , Rosenberg, S. , & Wolfe, R. (2010). The trauma of psychosis: Posttraumatic stress disorder and recent onset psychosis. Schizophrenia Research, 116(2–3), 217–227. 10.1016/j.schres.2009.10.025 19939633

[mpr2005-bib-0039] National Institute for Health and Clinical Excellence . (2018). Post‐traumatic stress disorder. Nice.org.uk. Retrieved November 30, 2021, from https://www.nice.org.uk/guidance/NG116

[mpr2005-bib-0040] OCEBM Levels of Evidence Working Group . (2011). The Oxford levels of evidence 2. Cebm.ox.ac.uk. Retrieved November 30, 2021, from https://www.cebm.ox.ac.uk/resources/levels‐of‐evidence/ocebm‐levels‐of‐evidence

[mpr2005-bib-0041] Okkels, N. , Trabjerg, B. , Arendt, M. , & Pedersen, C. (2016). Traumatic stress disorders and risk of subsequent schizophrenia spectrum disorder or bipolar disorder: A nationwide cohort study. Schizophrenia Bulletin, 43(1), 180–186. 10.1093/schbul/sbw082 27245172 PMC5216852

[mpr2005-bib-0042] Onyeama, C. , Vitale, K. , Cochran, K. , & Onyeama, G. (2011). Visual diagnosis: Swelling and redness of the fourth toe in a 3‐month‐old infant. Pediatric Review, 32(6), 253–255. 10.1542/pir.32-6-253 21632877

[mpr2005-bib-0043] Ouzzani, M. , Hammady, H. , Fedorowicz, Z. , & Elmagarmid, A. (2016). Rayyan—A web and mobile app for systematic reviews. Systematic Reviews, 5(1), 210. 10.1186/s13643-016-0384-4 27919275 PMC5139140

[mpr2005-bib-0044] Paulik, G. , Steel, C. , & Arntz, A. (2019). Imagery rescripting for the treatment of trauma in voice hearers: A case series. Behavioural and Cognitive Psychotherapy, 47(6), 709–725. 10.1017/s1352465819000237 30975230

[mpr2005-bib-0045] Peach, N. , Alvarez‐Jimenez, M. , Cropper, S. , Sun, P. , & Bendall, S. (2018). Testing models of post‐traumatic intrusions, trauma‐related beliefs, hallucinations, and delusions in a first episode psychosis sample. British Journal of Clinical Psychology, 58(2), 154–172. 10.1111/bjc.12206 30421797

[mpr2005-bib-0046] Peach, N. , Alvarez‐Jimenez, M. , Cropper, S. , Sun, P. , Halpin, E. , O’Connell, J. , & Bendall, S. (2020). Trauma and the content of hallucinations and post‐traumatic intrusions in first‐episode psychosis. Psychology and Psychotherapy: Theory, Research and Practice, 94(S2), 223–241. 10.1111/papt.12273 32154644

[mpr2005-bib-0047] Peters, E. (2020). STAR (Study of Trauma And Recovery): A trial of trauma‐focused psychological therapy for psychosis. Identification No. ISRCTN93382525. Isrctn.com. Retrieved December 2, 2021, from https://www.isrctn.com/ISRCTN93382525

[mpr2005-bib-0048] Saha, S. , Chant, D. , & McGrath, J. (2007). A systematic review of mortality in schizophrenia. Archives of General Psychiatry, 64(10), 1123. 10.1001/archpsyc.64.10.1123 17909124

[mpr2005-bib-0049] Schnurr, P. (2017). Focusing on trauma‐focused psychotherapy for posttraumatic stress disorder. Current Opinion in Psychology, 14, 56–60. 10.1016/j.copsyc.2016.11.005 28813321

[mpr2005-bib-0050] Shapiro, F. (2001). Eye movement desensitization and reprocessing (EMDR) therapy. Guilford Publications.

[mpr2005-bib-0051] Sin, J. , & Spain, D. (2016). Psychological interventions for trauma in individuals who have psychosis: A systematic review and meta‐analysis. Psychosis, 9(1), 67–81. 10.1080/17522439.2016.1167946

[mpr2005-bib-0052] Slotema, C. , van den Berg, D. , Driessen, A. , Wilhelmus, B. , & Franken, I. (2019). Feasibility of EMDR for posttraumatic stress disorder in patients with personality disorders: A pilot study. European Journal of Psychotraumatology, 10(1), 1614822. 10.1080/20008198.2019.1614822 31164968 PMC6534227

[mpr2005-bib-0053] Steel, C. (2015). Hallucinations as a trauma‐based memory: Implications for psychological interventions. Frontiers in Psychology, 6. 10.3389/fpsyg.2015.01262 PMC4569972 26441698

[mpr2005-bib-0054] Steel, C. , Hardy, A. , Smith, B. , Wykes, T. , Rose, S. , Enright, S. , Hardcastle, M. , Landau, S. , Baksh, M. F. , Gottlieb, J. D. , Rose, D. , & Mueser, K. T. (2016). Cognitive–behaviour therapy for post‐traumatic stress in schizophrenia. A randomized controlled trial. Psychological Medicine, 47(1), 43–51. 10.1017/s0033291716002117 27650432

[mpr2005-bib-0055] Sterne, J. , Savović, J. , Page, M. , Elbers, R. G. , Blencowe, N. S. , Boutron, I. , Cates, C. J. , Cheng, H. Y. , Corbett, M. S. , Eldridge, S. M. , Emberson, J. R. , Hernán, M. A. , Hopewell, S. , Hróbjartsson, A. , Junqueira, D. R. , Jüni, P. , Kirkham, J. J. , Lasserson, T. , Li, T. , …, & Higgins, J. P. T. (2019). RoB 2: A revised tool for assessing risk of bias in randomised trials. BMJ, l4898. 10.1136/bmj.l4898 31462531

[mpr2005-bib-0056] Strous, R. , Weiss, M. , Felsen, I. , Finkel, B. , Melamed, Y. , Bleich, A. , Kotler, M. , & Laub, D. (2005). Video testimony of long‐term hospitalized psychiatrically ill Holocaust survivors. American Journal of Psychiatry, 162(12), 2287–2294. 10.1176/appi.ajp.162.12.2287 16330592

[mpr2005-bib-0057] Swan, S. , Keen, N. , Reynolds, N. , & Onwumere, J. (2017). Psychological interventions for post‐traumatic stress symptoms in psychosis: A systematic review of outcomes. Frontiers in Psychology, 8. 10.3389/fpsyg.2017.00341 PMC5348513 28352239

[mpr2005-bib-0058] The EndNote Team . (2013). Endnote. Clarivate.

[mpr2005-bib-0059] The Lancet Psychiatry . (2020). A good enough measure. The Lancet Psychiatry, 7(10), 825. 10.1016/s2215-0366(20)30395-3 32949506

[mpr2005-bib-0060] Trappler, B. , & Newville, H. (2007). Trauma healing via cognitive behavior therapy in chronically hospitalized patients. Psychiatric Quarterly, 78(4), 317–325. 10.1007/s11126-007-9049-8 17924190

[mpr2005-bib-0061] Turner, S. , Beidel, D. , & Frueh, B. (2005). Multicomponent behavioral treatment for chronic combat‐related posttraumatic stress disorder. Behavior Modification, 29(1), 39–69. 10.1177/0145445504270872 15557478

[mpr2005-bib-0062] Valiente‐Gómez, A. , Pujol, N. , Moreno‐Alcázar, A. , Radua, J. , Monteagudo‐Gimeno, E. , Gardoki‐Souto, I. , Hogg, B. , Álvarez, M. J. , Safont, G. , Lupo, W. , Pérez, V. , & Amann, B. L. (2020). A multicenter phase II RCT to compare the effectiveness of EMDR versus TAU in patients with a first‐episode psychosis and psychological trauma: A protocol design. Frontiers in Psychiatry, 10, 1023. 10.3389/fpsyt.2019.01023 32116827 PMC7014965

[mpr2005-bib-0063] van den Berg, D. , de Bont, P. , van der Vleugel, B. , de Roos, C. , de Jongh, A. , van Minnen, A. , & van der Gaag, M. (2015). Trauma‐focused treatment in PTSD patients with psychosis: Symptom exacerbation, adverse events, and revictimization. Schizophrenia Bulletin, 42(3), 693–702. 10.1093/schbul/sbv172 26609122 PMC4838096

[mpr2005-bib-0064] van den Berg, D. , de Bont, P. , van der Vleugel, B. , de Roos, C. , de Jongh, A. , van Minnen, A. , & van der Gaag, M. (2018). Long‐term outcomes of trauma‐focused treatment in psychosis. The British Journal of Psychiatry, 212(3), 180–182. 10.1192/bjp.2017.30 29436320

[mpr2005-bib-0065] van den Berg, D. , & van der Gaag, M. (2012). Treating trauma in psychosis with EMDR: A pilot study. Journal of Behavior Therapy and Experimental Psychiatry, 43(1), 664–671. 10.1016/j.jbtep.2011.09.011 21963888

[mpr2005-bib-0066] Vila‐Badia, R. , Butjosa, A. , Del Cacho, N. , Serra‐Arumí, C. , Esteban‐Sanjusto, M. , Ochoa, S. , & Usall, J. (2021). Types, prevalence and gender differences of childhood trauma in first‐episode psychosis. What is the evidence that childhood trauma is related to symptoms and functional outcomes in first episode psychosis? A systematic review. Schizophrenia Research, 228, 159–179. 10.1016/j.schres.2020.11.047 33434728

[mpr2005-bib-0067] Ward‐Brown, J. , Keane, D. , Bhutani, G. , Malkin, D. , Sellwood, B. , & Varese, F. (2018). TF‐CBT and EMDR for young people with trauma and first episode psychosis (using a phasic treatment approach): Two early intervention service case studies. The Cognitive Behaviour Therapist, 11, e17. 10.1017/s1754470x18000193

[mpr2005-bib-0068] Yasar, A. , Kiraz, S. , Usta, D. , Abamor, A. , Kavakci, O. , & Zengin Eroglu, M. (2017). Eye movement desensitization and reprocessing (EMDR) therapy on a patient with schizophrenia and clinical effects: A case study. Turkish Journal of Psychiatry, 29(2), 138. 10.5080/u18328 30215843

[mpr2005-bib-0069] Yuen, K. , Harrigan, S. , Mackinnon, A. , Harris, M. G. , Yuen, H. P. , Henry, L. P. , Jackson, H. J. , Herrman, H. , & McGorry, P. D. (2014). Long‐term follow‐up of all‐cause and unnatural death in young people with first‐episode psychosis. Schizophrenia Research, 159(1), 70–75. 10.1016/j.schres.2014.07.042 25151199

