# Supplementary material for: The effectiveness and tolerability of trauma‐focused psychotherapies for psychotic symptoms: A systematic review of trauma‐focused psychotherapies
Source: Int J Methods Psychiatr Res. 2024 Mar 5;33(1):e2005. doi: 10.1002/mpr.2005 (PMC10914124; doi:10.1002/mpr.2005)
Supplement: Supplementary file 1 — Supporting Information S1 [file MPR-33-e2005-s001.docx]

**The effectiveness and tolerability of trauma-focussed psychotherapies for psychotic symptoms: a systematic review: supplement**

Systematic review of trauma-focused psychotherapies: supplement

1. Jordan Reid^a^, ucjteid@ucl.ac.uk
2. Charles Cole^b^, charles.cole.15@ucl.ac.uk
3. Nabeela Malik^c^, nabeela.malik1@nhs.net
4. Vaughan Bell^b^, vaughan.bell@ucl.ac.uk
5. Michael Bloomfield^a^, m.bloomfield@ucl.ac.uk (Corresponding author)

Corresponding author:
m.bloomfield@ucl.ac.uk, +44 (0)20 3549 5872
Translational Psychiatry Research Group, Research Department of Mental Health Neuroscience, Division of Psychiatry, Institute of Mental Health, UCL

^a^University College London, Translational Psychiatry Research Group, Research Department of Mental Health Neuroscience, Division of Psychiatry, Institute of Mental Health, W1T 7NF

^b^University College London, Department of Clinical, Educational and Health Psychology, 1-19 Torrington Place, London WC1E 7HB

^c^University of Hertfordshire, College Lane, Hatfield, AL10

**Supplementary Discussion**

Further to the discussion provided in our review, additional factors we identified that may be relevant to the question of efficacy of trauma-focused psychotherapies (TFPT) for psychotic symptoms include the use of psychological formulations, severity of psychotic symptoms, the dose of TFPT, and combination treatment with pharmacotherapy.

A formulation (i.e., case conceptualisation) was used in the two studies with the greatest impact on psychotic symptoms,^1,2^ with the latter study indicating greater effect sizes for participants whose experience of trauma could be understood by the patient as related to their psychotic symptoms. Various researchers have recommended the use of approaches that target PTSD and psychotic symptoms in an integrated fashion;^3,4^ a formulation that incorporates an understanding of the potential relationship between experiences of trauma and psychotic symptoms is a key aspect of this process.

It is possible that psychotic symptom severity may also play a moderating role in response to TFPT. Overall, the included studies conducted with inpatient samples appeared to show a smaller effect of TFPT for reducing psychotic symptoms compared to out-patient settings.^1,5^ This is in line with the literature in the area, which indicates that the effectiveness of psychological therapies could be impacted by the course and severity of the psychotic symptoms.^6^ There are several reasons why patients at an acute stage of psychosis requiring in-patient treatment may receive less benefit from TFPT including being too unwell when acutely psychotic to engage with and take benefit from TFPT. However, the most recent guidelines from the International Society of Traumatic Stress Studies (ISTSS)^7^ no longer recommend a phase-based approach where time is given to ‘stabilisation’ work, intended to improve emotional, social, and psychological coping abilities, before moving onto trauma-focused work. This was influenced by research which concluded that delaying trauma-focused treatment could be demoralising and negatively impact self-efficacy.^8^ Further work may be needed to understand the role of potential role of stabilisation for TFPT in patients with psychosis.

Some studies which did not find a significant impact of TFPT on psychotic symptoms did not require a ‘minimum’ level of psychotic symptoms in their participants.^9,10^ Whilst it is possible that these findings are due to ceiling and floor effects, taken together, it is possible that there may be a therapeutic “window” of intervention for TFPT, where psychotic symptoms are severe enough to be problematic but not to the point of requiring admission into an inpatient unit.

Differences in the dose of TFPT across studies also appear relevant. Regarding the studies investigating TICBT or TFCBT, the minimum number of sessions considered sufficient exposure to the intervention across protocols was six. This is comparable with the number of sessions provided in a recent cognitive behavioural therapy for psychosis (CBTp) meta-analysis (minimum 4, mean 14·75)^11^ and a recent trial of a “feeling safe” programme for persecutory delusions (minimum 6, mean 19·1)^12^ Nevertheless, it has been suggested that 25 sessions are the optimal dose for CBTp;^13^ much more than the majority of studies in this review. Regarding EMDR studies, we identified a greater variation in how many sessions were offered, from three^5^ to fifteen.^9^ Whilst the number of EMDR sessions that are required to impact upon psychotic symptoms is not currently known, it is likely that this patient group requires a longer course of treatment given the need for enhanced engagement, socialisation to the model, and the targeting of psychotic and post-traumatic symptoms. In support of this, one study which found a positive impact on psychotic symptoms provided up to 66 sessions.^14^

Finally, it is important to note that, when reported upon, each included study noted their participants were concurrently in receipt of TAU from their mental health service, often including prescription of psychiatric medication. It is therefore not possible at this stage to say TFPT could be effective without continuing multidisciplinary input and medication, and evidence has shown more generally that psychotherapy in combination with medication is the most effective for patients with psychosis.^15^

Based on the above we identify here clinical recommendations in addition to those included in our review. Therapists should consider exploring and formulating potential thematic links between DT and psychotic symptoms. This would enable the therapist to provide a rationale for targeting a range of psychotic symptoms, such as exposure to memories that may be ‘echoes’ of a past trauma, or by providing less distressing alternatives to post-traumatic or delusional beliefs. A shared formulation also helps to normalise a person with psychosis’ experiences and to improve the therapeutic relationship, both of which are considered of particular importance in working with people with psychosis^16^ (conditional recommendation).

Due to the impact of TFPT dose and the value of formulation, it is recommended to offer a sufficient number of sessions to patients. This review is not able to ascertain the appropriate dose of TFPT for people with psychosis. A greater number of sessions would also help to accommodate for cognitive processing difficulties associated with psychosis^17^ and it is clinically reasonable to anticipate that greater severity of psychosis may require more time to build a therapeutic relationship, motivate the patient to change unpick trauma histories, understand their relationships to current symptoms and to progress into treatment (strong recommendation).

Further well-controlled research is needed to investigate the efficacy of TFPT for psychotic symptoms. We recommend that treatment protocols investigate the factors identified in our review such as formulation work to link post-traumatic and psychotic symptoms, the use of exposure on appropriate trauma targets agreed through formulation work, and appropriate treatment length to allow sufficient time for formulation, socialisation to the model and engagement.

Research would benefit from evaluating the nature of the relationship between post-traumatic stress (PTS) and psychotic symptoms. Large, controlled studies with frequent and regular measurements will be vital to investigate temporal relationships between PTS and psychotic symptoms.

Future research should also address nosological issues regarding whether psychosis in the context of trauma could be considered a psychotic form of PTSD, or PTSD with psychotic features. Such work would inform any modifications to diagnostic systems such as the ICD12.

In summary, factors that may alter the effectiveness of TFPT for psychotic symptoms include the use of psychological formulations, the severity of psychotic symptoms, and the dose of TPFT. Whilst each of these factors requires further empirical investigation, clinicians should consider these factors when collaboratively developing psychological treatment plans with their patients.

**Supplement A**

**Full List of Systematic Review Search Terms by Database**

| **Ovid MEDLINE(R) and Epub Ahead of Print, In-Process & Other Non-Indexed Citations and Daily**  1946 to 2020 | | |
| --- | --- | --- |
| **Search 1:**  Psychotic symptoms | **Search 2:**  Trauma-focussed | **Search 3:**  Psychotherapy |
| exp "schizophrenia spectrum and other psychotic disorders"/ OR Borderline Personality Disorder/ OR exp "Bipolar and Related Disorders"/ OR psychotic.ab,ti OR hallucinat*.ab,ti OR delus*.ab,ti OR paranoi*.ab,ti OR voice*.ab,ti OR intrusi*.ab,ti OR "ultra high risk".ab,ti OR UHR.ab,ti OR "at risk mental state".ab,ti | exp "Trauma and Stressor Related Disorders"/ OR exp Crime Victims/ OR exp Bullying/ OR exp adverse childhood experiences/ or exp child abuse/ or exp physical abuse/ or exp rape/ or exp terrorism/ or exp torture/ OR exp Survivors/ OR "Trauma-focu?ed".ab,ti OR trauma*.ab,ti OR PTSD.ab,ti OR "post-traumatic stress".ab,ti OR CPTSD.ab,ti OR "complex trauma".ab,ti OR neglect*.ab,ti OR maltreatment.ab,ti OR bully*.ab,ti OR victim*.ab,ti OR reprocessing.ab,ti | exp Psychotherapy/ OR "psychological therapy".ab,ti OR "Cognitive Behavi?r* Therap*".ab,ti OR CBT.ab,ti OR "Eye Movement Desensiti?ation Reprocessing".ab,ti OR EMDR.ab,ti OR "Narrative Exposure Therapy".ab,ti OR NET.ab,ti OR "Contextual Processing Theory".ab,ti OR "Cognitive Processing Therapy".ab,ti or CPT.ab,ti OR "Exposure Therapy".ab,ti OR "Systemic Therapy".ab,ti OR counselling.ab,ti OR "Behavi?ral Therapy".ab,ti OR "Cognitive Therapy".ab,ti OR psychoanaly*.ab,ti OR psychodynamic.ab,ti |
| **Search 4:**  Search 1 AND Search 2 AND Search 3  Limited to English and Journal Article | | |

| **APA PsycInfo**  1806 to 2020 | | |
| --- | --- | --- |
| **Search 1:**  Psychotic symptoms | **Search 2:**  Trauma-focussed | **Search 3:**  Psychotherapy |
| exp psychosis/ OR bipolar disorder/ OR borderline personality disorder/ OR psychotic.ab,ti OR hallucinat*.ab,ti OR delus*.ab,ti OR paranoi*.ab,ti OR voice*.ab,ti OR intrusi*.ab,ti OR "ultra high risk".ab,ti OR UHR.ab,ti OR "at risk mental state".ab,ti | "emotional trauma"/ OR exp injuries/ OR "post-traumatic stress"/ OR "traumatic loss"/ OR exp adversity/ OR exp disasters/ OR exp accidents/ OR exp "stress and trauma related disorders"/ OR exp crime victims/ OR exp bullying/ OR exp Child Abuse/ or exp Emotional Abuse/ or exp Physical Abuse/ or exp Sexual Abuse/ OR exp survivors/ OR "Trauma-focu?ed".ab,ti OR trauma*.ab,ti OR PTSD.ab,ti OR "post-traumatic stress".ab,ti OR CPTSD.ab,ti OR "complex trauma".ab,ti OR neglect*.ab,ti OR maltreatment.ab,ti OR bully*.ab,ti OR victim*.ab,ti OR reprocessing.ab,ti | exp psychotherapy/ OR "psychological therapy".ab,ti OR "Cognitive Behavi?r* Therap*".ab,ti OR CBT.ab,ti OR "Eye Movement Desensiti?ation Reprocessing".ab,ti OR EMDR.ab,ti OR "Narrative Exposure Therapy".ab,ti OR NET.ab,ti OR "Contextual Processing Theory".ab,ti OR "Cognitive Processing Therapy".ab,ti or CPT.ab,ti OR "Exposure Therapy".ab,ti OR "Systemic Therapy".ab,ti OR counselling.ab,ti OR "Behavi?ral Therapy".ab,ti OR "Cognitive Therapy".ab,ti OR psychoanaly*.ab,ti OR psychodynamic.ab,ti |
| **Search 4:**  Search 1 AND Search 2 AND Search 3  Limited to English and Peer-Reviewed Journal | | |

| **PTSDPubs**  1945 to 2020 | | |
| --- | --- | --- |
| **Search 1:**  Psychotic symptoms | **Search 2:**  Trauma-focussed | **Search 3:**  Psychotherapy |
| MAINSUBJECT.EXACT.EXPLODE(“Psychotic Disorders”) OR MAINSUBJECT.EXACT.EXPLODE(“Bipolar Disorders”) OR MAINSUBJECT.EXACT.EXPLODE(“Borderline Personality Disorder”) OR ab,ti(psychotic OR hallucinat* OR delus* OR paranoi* OR voice* OR intrusi* OR "ultra high risk" OR UHR OR "at risk mental state") | MAINSUBJECT.EXACT.EXPLODE("PTSD") OR MAINSUBJECT.EXACT.EXPLODE(“Stressors”) OR MAINSUBJECT.EXACT.EXPLODE(“Survivors”) OR ab,ti("Trauma-focu?ed" OR trauma* OR PTSD OR "post-traumatic stress" OR CPTSD OR "complex trauma" OR neglect* OR maltreatment OR bully* OR victim* OR reprocessing) | MAINSUBJECT.EXACT.EXPLODE(“Psychotherapy”) OR ab,ti("psychological therapy" OR "Cognitive Behavi?r* Therap*" OR CBT OR "Eye Movement Desensiti?ation Reprocessing" OR EMDR OR "Narrative Exposure Therapy" OR NET OR "Contextual Processing Theory" OR "Cognitive Processing Therapy" or CPT OR "Exposure Therapy" OR "Systemic Therapy" OR counselling OR "Behavi?ral Therapy" OR "Cognitive Therapy" OR psychoanaly* OR psychodynamic) |
| **Search 4:**  Search 1 AND Search 2 AND Search 3  Limited to English and Scholarly Journals | | |

**Supplement B**

**Formulae Regarding the Calculation of Hedges’ g**

For between-subjects designs where Cohen’s d was reported, Hedges’ g was calculated using the J-correction (Hedges & Olkin, 1985):
**B.1**
**
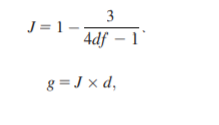
**

Where this was not reported, where possible, Hedges’ g was calculated manually from reported standard deviations, means and n. If standard deviation was not reported, then these were calculated from data that were available, such as confidence intervals, standard errors, t-values or p-values, following Cochrane Handbook procedures (Higgins & Green, 2011).

For within-subjects designs where Cohen’s d was reported, Hedges’ g was calculated using the J-correction (Hedges & Olkin, 1985):
**B.2**


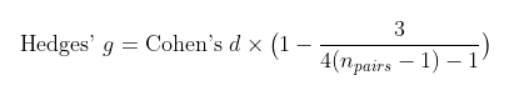


Where this was not reported, where possible, an effect size was calculated manually. To do so, an estimate of the correlation between pre- and post-scores is required to account for the violation of independence. Given this is rarely reported, an estimate of 0.6 was used based on the median within-group correlation calculated from 811 pre-post clinical trial arms (Balk et al., 2012). The following formula was used:

**B.3**


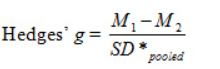


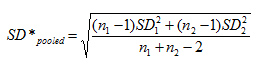


In cases where further assumptions of Cohen’s d and Hedges’ g are violated (such as the requirement for a normal distribution and to be based on means), the effect size r has been calculated from z where necessary using the following formula:
**B.4**

r = z/√N.

**Supplement C**

**Case Series Quality Appraisal Questions by Number**

**Quality Appraisal Checklist for Case Series Studies
and Instructions for Use***

| **Study objective** | |
| --- | --- |
| **1.** | **Was the hypothesis/aim/objective of the study clearly stated?**  **Yes**: The hypothesis/aim/objective of the study was clearly reported (includes patients, intervention and outcome).  **Partial**: Only one or two components (patients, intervention, or outcome) were included.  **No**: The hypothesis/aim/objective was not reported. |
| **Study design** | |
| **2.** | **Was the study conducted prospectively?**  **Yes:** It was clearly stated that the study was conducted prospectively.  **Unclear**: Unclear or no information was provided.  **No:** The study clearly stated it was a retrospective study. |
| **3.** | **Were the cases collected in more than one centre?**  **Yes**: Cases were collected in more than one centre (multicentre study).  **Unclear**: Unclear where the patients came from.  **No**: Cases were collected from one centre. |
| **4.** | **Were patients recruited consecutively?**  **Yes**: There was a clear statement or it was clear from the context that the patients were recruited consecutively; or the study stated that all eligible patients were recruited.  **Unclear**: No information was provided about the method used to recruit patients in the study.  **No**: The study clearly stated that patients were not recruited consecutively; or the patients were recruited based on other criteria such as access to intervention determined by the distance or availability of resources. |
| **Study population** | |
| **5.** | **Were the characteristics of the patients included in the study described?**  **Yes**: All of the most relevant characteristics of the patients were reported (for example, number, age, gender, ethnicity, severity of disease/condition, comorbidity, or etiology).  **Partial**: Some, but not all, of the most relevant characteristics were reported.  **No**: Only the number of patients was reported.  *Note: Assessor(s) should decide which aspects are important before using the checklist.* |
| **6.** | **Were the eligibility criteria (i.e. inclusion and exclusion criteria) for entry into the study clearly stated?**  **Yes**: Both inclusion and exclusion criteria were reported.  **Partial**: Either the inclusion or exclusion criteria were reported.  **No**: Neither inclusion nor exclusion criteria were reported.  *Note: Assessor(s) should decide which aspects are important before using the checklist.* |
| **7.** | **Did patients enter the study at a similar point in the disease?**  **Yes**: It was clear from the baseline data presented in the study (for example, tables of patients’ characterises) that the majority (at least 80%) of patients entered the study at a similar point in terms of the duration and severity of the disease/condition and the presence of co-morbidities/complications.  **Unclear**: There was no baseline information on patients’ characteristics to make a judgment.  **No**: There was a wide range in the severity of the disease/condition and co-morbidities/complications in patients at baseline.  *Note: Assessor(s) should decide which aspects are important before using the checklist.* *It might be useful to discuss with specialists to determine the most important aspects that should be considered.* |
| **Intervention and co-intervention** | |
| **8.** | **Was the intervention of interest clearly described?**  **Yes**: All of the most relevant characteristics of the intervention were reported (for example, dosage, frequency or duration of intervention, administration methods, technical parameters, or characteristics of a device).  **Partial**: Some, but not all, of the most relevant characteristics were reported.  **No**: Only the name of the intervention was reported.  *Note: Assessor(s) should decide which aspects are important before using the checklist.* |
| **9.** | **Were additional interventions (co-interventions) clearly described?**  **Yes**: All of the most relevant characteristics of the co-intervention(s) were reported (for example, different type, dosage, frequency of administration, or duration); or the study clearly stated that a co-intervention was not administered for clinical reasons.  **Partial**: Some, but not all, of the most relevant characteristics of the co-intervention were reported.  **No**: No information about co-intervention(s) was provided; or only the name(s) of the co-intervention(s) were mentioned.  *Note: Assessor(s) should decide which aspects are important before using the checklist.* |
| **Outcome measures** | |
| **10.** | **Were relevant outcome measures established a priori?**  **Yes**: All relevant outcome measures were stated in the introduction or methods section.  **Partial**: Some, but not all, of the relevant outcome measures were stated in the introduction or method section.  **No**: None of the relevant outcome measures were stated in the introduction or method section. |
| **11.** | **Were outcome assessors blinded to the intervention that patients received?**  **Yes**: The relevant outcomes were assessed by individuals who were not aware of the intervention. Answer yes when blinding is not applicable or is unnecessary (for example, mortality).  **Unclear**: The study did not report whether the outcome assessors were aware of the intervention.  **No**: It was clearly stated or obvious from the context that the relevant outcomes were analyzed by individuals who were aware of the intervention provided to patients. |
| **12.** | **Were the relevant outcomes measured using appropriate objective/subjective methods?**  **Yes**: All relevant outcomes were measured with appropriate methods. These measures can be objective (for example, gold standard tests or standardized clinical tests), subjective (for example, self-administered questionnaires, standardized forms, or patient symptoms interview forms), or both.  **Partial**: Some, but not all, relevant outcomes were measured with appropriate methods.  **No**: The methods used to measure the relevant outcomes were inappropriate.  *Note: Assessor(s) should decide which methods are appropriate before using the checklist.* |
| **13.** | **Were the relevant outcome measures made before and after the intervention?**  **Yes**: The relevant outcome measures were made pre- and post-intervention; or the baseline measurements were not possible (for example, death).  **Unclear**: The study did not report when the outcome measures were made.  **No**: The outcome measures were only made post-intervention. |
| **Statistical analysis** | |
| **14.** | **Were the statistical tests used to assess the relevant outcomes appropriate?**  **Yes**: The statistical tests were used appropriately (for example, parametric test for normally distributed population vs. nonparametric test for non-Gaussian population). Answer yes if no statistical analysis was performed and reasons for this were stated.  **Unclear**: The statistical tests were not described in the methods section of the study.  **No**: The statistical tests used were inappropriate.  *Note:* *Assessor(s) should decide which statistical tests are appropriate before using the checklist. Seek expert assistance if necessary.* |
| **Results and conclusions** | |
| **15.** | **Was follow-up long enough for important events and outcomes to occur?**  **Yes**: It was clear from the information provided that the follow-up period was long enough for the majority (at least 80%) of patients, to allow for important events and outcomes (for example, changes in clinical status, adverse events) to occur.  **Unclear**: The length of follow-up was not clearly reported.  **No**: It is clear from the information provided that the follow-up period was not long enough to allow for important events and outcomes to occur.  *Note:* *Assessor(s) should define the appropriate duration of follow-up for each outcome of interest (for example, short-term and long-term adverse events).* |
| **16.** | **Were losses to follow-up reported?**  **Yes**: The number or proportion of patients lost to follow-up was clearly reported; the authors reported outcome results on all patients initially included; or the number lost to follow-up can be subtracted from the number of patients enrolled and the number of patients included in the final analysis.  **Unclear**: There was a discrepancy between the number or proportion of patients reported in tables, figures, and text.  **No**: The number or proportion of patients lost to follow-up was not reported. |
| **17.** | **Did the study provided estimates of random variability in the data analysis of relevant outcomes?**  **Yes**: The estimates of the random variability (for example, standard error, standard deviation, confidence interval for normally distributed data or range and interquartile range for non-normally distributed data) were reported for all of the relevant outcomes or could be calculated from the raw data presented in the study.  **Partial**: The estimates of the random variability were reported for some, but not all of the relevant outcomes.  **No**: The estimates of the random variability were not reported for any of the relevant outcomes. |
| **18.** | **Were the adverse events reported?**  **Yes**: The undesirable or unwanted events during the study period or within a pre-specified time period were reported; or the absence of adverse event(s) was mentioned in the study.  **Partial**: Some, but not all, important adverse events were reported.  **No**: There was no statement about the presence or absence of adverse events.  *Note:* *Assessor(s) should decide which adverse events are most important. Seek clinical expert assistance if necessary.* |
| **19.** | **Were the conclusions of the study supported by the results?**  **Yes**: The conclusions of the study were supported by the evidence presented in the results and discussion sections.  **Unclear**: Unclear conclusion statement that makes it difficult to link the presented evidence to conclusions.  **No**: The conclusions were not supported by the evidence presented in the results and discussion sections. |
| **Competing interests and sources of support** | |
| **20.** | **Were both competing interests and sources of support for the study reported?**  **Yes**: Both competing interests and sources of support (financial or other) received for the study were reported; or the absence of any competing interest and source of support was acknowledged.  **Partial**: Either the competing interest or source of support was reported.  **No**: Neither competing interests nor sources of support were reported. |

**References**

1. de Bont P, van den Berg D, van der Vleugel B et al. Prolonged exposure and EMDR for PTSDv. a PTSD waiting-list condition: effects on symptoms of psychosis, depression and social functioning in patients with chronic psychotic disorders. *Psychol Med*. 2016;46(11):2411-2421. doi:10.1017/s0033291716001094

2. Paulik G, Steel C, Arntz A. Imagery rescripting for the treatment of trauma in voice hearers: a case series. *Behav Cogn Psychother*. 2019;47(6):709-725. doi:10.1017/s1352465819000237

3. Keen N, Hunter E, Peters E. Integrated Trauma-Focused Cognitive-Behavioural Therapy for Post-traumatic Stress and Psychotic Symptoms: A Case-Series Study Using Imaginal Reprocessing Strategies. *Front Psychiatry*. 2017;8(8):92. doi:10.3389/fpsyt.2017.00092

4. Peach N, Alvarez‐Jimenez M, Cropper S, Sun P, Bendall S. Testing models of post‐traumatic intrusions, trauma‐related beliefs, hallucinations, and delusions in a first episode psychosis sample. *British Journal of Clinical Psychology*. 2018;58(2):154-172. doi:10.1111/bjc.12206

5. Kim D, Choi J, Kim S, Oh D, Park S, Lee S. A Pilot Study of Brief Eye Movement Desensitization and Reprocessing(EMDR) for Treatment of Acute Phase Schizophrenia. *Korean Journal of Biological Psychiatry*. 2010;17:94-102.

6. Munro S, Baker J, Playle J. Cognitive behaviour therapy within acute mental health care: A critical appraisal. *Int J Ment Health Nurs*. 2005;14(2):96-102. doi:10.1111/j.1440-0979.2005.00365.x

7. Bisson J, Berliner L, Cloitre M et al. The International Society for Traumatic Stress Studies New Guidelines for the Prevention and Treatment of Posttraumatic Stress Disorder: Methodology and Development Process. *J Trauma Stress*. 2019;32(4):475-483. doi:10.1002/jts.22421

8. De Jongh A, Resick P, Zoellner L et al. CRITICAL ANALYSIS OF THE CURRENT TREATMENT GUIDELINES FOR COMPLEX PTSD IN ADULTS. *Depress Anxiety*. 2016;33(5):359-369. doi:10.1002/da.22469

9. Slotema C, van den Berg D, Driessen A, Wilhelmus B, Franken I. Feasibility of EMDR for posttraumatic stress disorder in patients with personality disorders: a pilot study. *Eur J Psychotraumatol*. 2019;10(1):1614822. doi:10.1080/20008198.2019.1614822

10. de Bont P, van Minnen A, de Jongh A. Treating PTSD in Patients With Psychosis: A Within-Group Controlled Feasibility Study Examining the Efficacy and Safety of Evidence-Based PE and EMDR Protocols. *Behav Ther*. 2013;44(4):717-730. doi:10.1016/j.beth.2013.07.002

11. Turner D, Reijnders M, van der Gaag M et al. Efficacy and Moderators of Cognitive Behavioural Therapy for Psychosis Versus Other Psychological Interventions: An Individual-Participant Data Meta-Analysis. *Front Psychiatry*. 2020;11:402. doi:10.3389/fpsyt.2020.00402

12. Freeman D, Emsley R, Diamond R et al. Comparison of a theoretically driven cognitive therapy (the Feeling Safe Programme) with befriending for the treatment of persistent persecutory delusions: a parallel, single-blind, randomised controlled trial. *The Lancet Psychiatry*. 2021;8(8):696-707. doi:10.1016/s2215-0366(21)00158-9

13. Lincoln T, Jung E, Wiesjahn M, Schlier B. What is the minimal dose of cognitive behavior therapy for psychosis? An approximation using repeated assessments over 45 sessions. *European Psychiatry*. 2016;38:31-39. doi:10.1016/j.eurpsy.2016.05.004

14. Keen N, Hunter E, Peters E. Integrated Trauma-Focused Cognitive-Behavioural Therapy for Post-traumatic Stress and Psychotic Symptoms: A Case-Series Study Using Imaginal Reprocessing Strategies. *Front Psychiatry*. 2017;8:92. doi:10.3389/fpsyt.2017.00092

15. Cuijpers P, Sijbrandij M, Koole S, Andersson G, Beekman A, Reynolds C. Adding Psychotherapy to Antidepressant Medication in Depression and Anxiety Disorders: a Meta-Analysis. *FOCUS*. 2014;12(3):347-358. doi:10.1176/appi.focus.12.3.347

16. Morrison A, Renton J, Dunn H, Williams S, Bentall R. *Cognitive Therapy For Psychosis: A Formulation-Based Approach.*. London: Routledge; 2004.

17. Barch D, Sheffield J. Cognitive impairments in psychotic disorders: common mechanisms and measurement. *World Psychiatry*. 2014;13(3):224-232. doi:10.1002/wps.20145
