# Supplementary material for: The effectiveness and tolerability of trauma‐focused psychotherapies for psychotic symptoms: A systematic review of trauma‐focused psychotherapies
Source: Int J Methods Psychiatr Res. 2024 Mar 5;33(1):e2005. doi: 10.1002/mpr.2005 (PMC10914124; doi:10.1002/mpr.2005)
Supplement: Supplementary file 2 — Tables S1–S3 [file MPR-33-e2005-s002.docx]

| **Authors** | **Randomisation** | **Deviations from Intended Intervention** | **Missing Outcome Data** | **Measurement of Outcome** | **Selection of Report of Result** | **Overall** |
| --- | --- | --- | --- | --- | --- | --- |
| de Bont et al. (2016)^46^ | Low | Low | Some concerns | Low | Low | Some concerns |
| Mueser et al. (2015)^38^ | Low | Some concerns | Some concerns | Low | Low | Some concerns |
| Kim et al. (2010)^47^ | Some concerns | Low | Some concerns | Low | Low | Some concerns |
| Steel et al. (2017)^39^ | Low | Low | Some concerns | Low | Low | Some concerns |

***Supplementary Table 1:* RCT Quality Assessment**

| **Authors** | **1** | **2** | **3** | **4** | **5** | **6** | **7** | **8** | **9** | **10** | **11** | **12** | **13** | **14** | **15** | **16** | **17** | **18** | **19** | **20** |
| --- | --- | --- | --- | --- | --- | --- | --- | --- | --- | --- | --- | --- | --- | --- | --- | --- | --- | --- | --- | --- |
| de Bont et al. (2013)^51^ | Y | Y | N | Y | Y | Y | U | Y | Y | Y | N | Y | Y | Y | Y | Y | Y | Y | Y | P |
| Keen et al. (2017)^41^ | Y | Y | N | Y | Y | P | N | Y | Y | Y | U | Y | Y | Y | Y | Y | Y | Y | Y | P |
| Paulik et al. (2019)^53^ | Y | Y | N | Y | Y | Y | N | Y | Y | Y | N | Y | Y | Y | Y | Y | Y | Y | Y | Y |
| Slotema et al. (2019)^48^ | Y | Y | N | N | Y | Y | N | Y | Y | Y | U | Y | Y | Y | N | Y | Y | P | Y | Y |
| Van den Berg and Van der Gaag (2012)^49^ | N | Y | Y | U | Y | Y | U | Y | Y | Y | U | Y | Y | Y | N | Y | Y | Y | Y | P |
| Trappler and Newville (2007)^36^ | P | Y | N | N | N | P | U | Y | Y | Y | N | Y | Y | Y | N | Y | N | N | Y | N |
| Brand and Loewenstein (2014)^37^ | Y | Y | Y | N | Y | Y | N | N | Y | Y | N | Y | Y | Y | Y | Y | Y | N | Y | N |
| Strous et al. (2005)^54^ | Y | Y | Y | N | P | Y | U | Y | N | Y | N | Y | Y | Y | Y | Y | Y | Y | Y | P |

*Note.* Y = Yes. N = No. P = Partial. U = Unclear. Please see **Supplementary Materials** for the full list of quality appraisal questions by number.

***Supplementary Table 2:* Case Series Quality Assessment**

| **Authors** | **Demographics Described?** | **History Described?** | **Current Condition Described?** | **Diagnostic Tests/ Assessments Described?** | **Intervention Described?** | **Post-intervention Condition Described?** | **Adverse Events Described?** | **Takeaway Lessons Provided?** | **Overall Appraisal** |
| --- | --- | --- | --- | --- | --- | --- | --- | --- | --- |
| Arens (2014)^55^ | Yes | Yes | Yes | Yes | Yes | Yes | Yes | Yes | Include |
| Callcot et al. (2004)^42^ | Yes | No | Yes | No | Yes | Yes | No | Yes | Include |
| McCartney et al. (2019)^44^ | Yes | No | Yes | Yes | Yes | Yes | Yes | Yes | Include |
| Ward-Brown et al. (2018)^43^ | Yes | No | Yes | Yes | Yes | Yes | Yes | Yes | Include |
| Yaşar et al. (2018)^50^ | Yes | Yes | Yes | Yes | Yes | Yes | Yes | Yes | Include |

***Supplementary Table 3:* Case Report Quality Assessment**
